# Supplementary material for: The Role of Triazole and Glucose Moieties in Alkali Metal Cation Complexation by Lower-Rim Tertiary-Amide Calix[4]arene Derivatives
Source: Molecules. 2022 Jan 12;27(2):470. doi: 10.3390/molecules27020470 (PMC8780480; doi:10.3390/molecules27020470)
Supplement: Supplementary file 1 [file molecules-27-00470-s001.zip › molecules-1537473-supplementary.pdf]

The role of triazole and glucose moieties in alkali metal cation complexation by lower-rim tertiary-amide calix[4]arene derivatives

SUPPORTING INFORMATION

J. Požar*, M. Cvetnić, A. Usenik, N. Cindro, G. Horvat, K. Leko, M. Modrušan, V. Tomišić*

Department of Chemistry, Faculty of Science, University of Zagreb, Horvatovac 102a, 10000 Zagreb, Croatia

Contents

1. Complexation of alkali metal cations with compound **L** in methanol 2
2. Complexation of alkali metal cations with compound **L** in acetonitrile 5
3. Complexation of alkali metal cations with compound **L** in *N*,*N*-dimethylformamide 9
4. Complexation of alkali metal cations with compound **l** in methanol 11
5. Complexation of alkali metal cations with compound **l** in *N*,*N*-dimethylformamide 13
6. The solvation of receptors in studied solvents 15
7. Inclusion of solvent molecule into calixarene **L** 18
8. The solvent effect on the alkali metal complexation and comparison of **L** and **l** binding affinities 20
9. Molecular dynamics simulations: Structures of **L** and **l** in MeCN, MeOH, and DMF 23
10. Molecular dynamics simulations: Complexes of **L** and **l** in MeCN, MeOH and DMF 27
11. Molecular dynamics simulations: graphical summation of the results obtained for alkali metal complexes with glycocalixarene **l** 54

1. Complexation of alkali metal cations with compound L in methanol

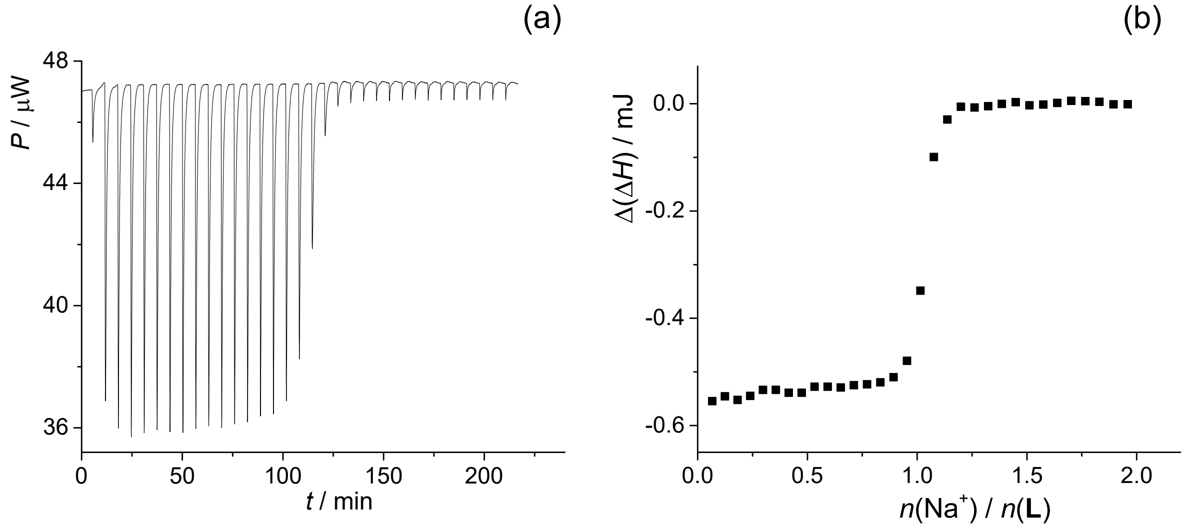

Figure S1. a) Microcalorimetric titration of **L** (c = 1.10 × 10^{-4} mol dm^{-3}, V = 1.43 mL) with $NaClO_4$ (c = 1.49 × 10^{-3} mol dm^{-3}) in methanol at 25 °C; b) Dependence of successive enthalpy change on $n(NaClO_4)$ / n(**L**) ratio.

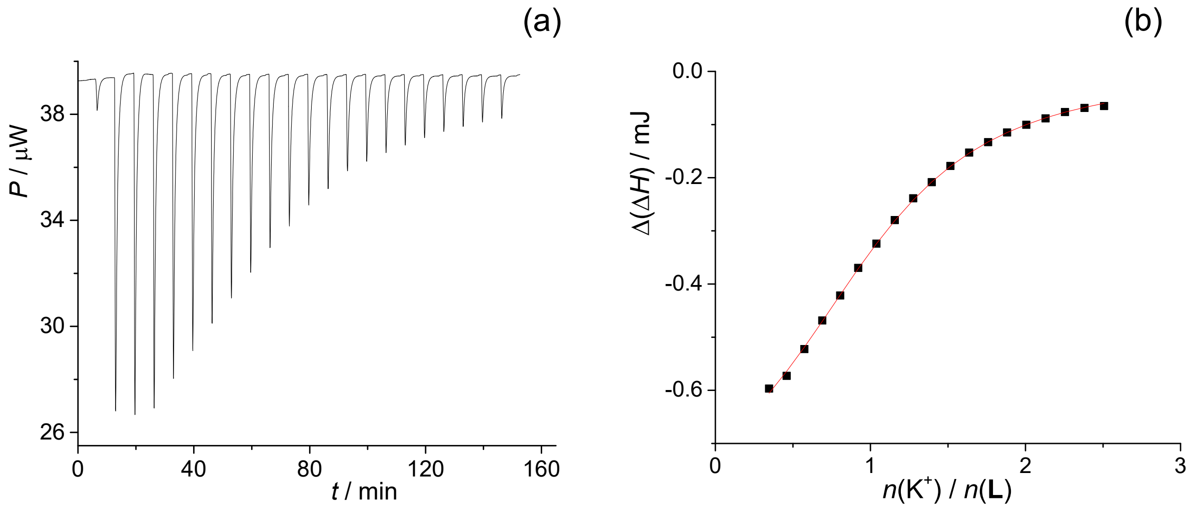

Figure S2. a) Microcalorimetric titration of **L** (c = 1.21 × 10^{-4} mol dm^{-3}, V = 1.43 mL) with $KClO_4$ (c = 1.91 × 10^{-3} mol dm^{-3}) in methanol at 25 °C; b) Dependence of successive enthalpy change on $n(KClO_4)$ / n(**L**) ratio. ■ experimental; — calculated.

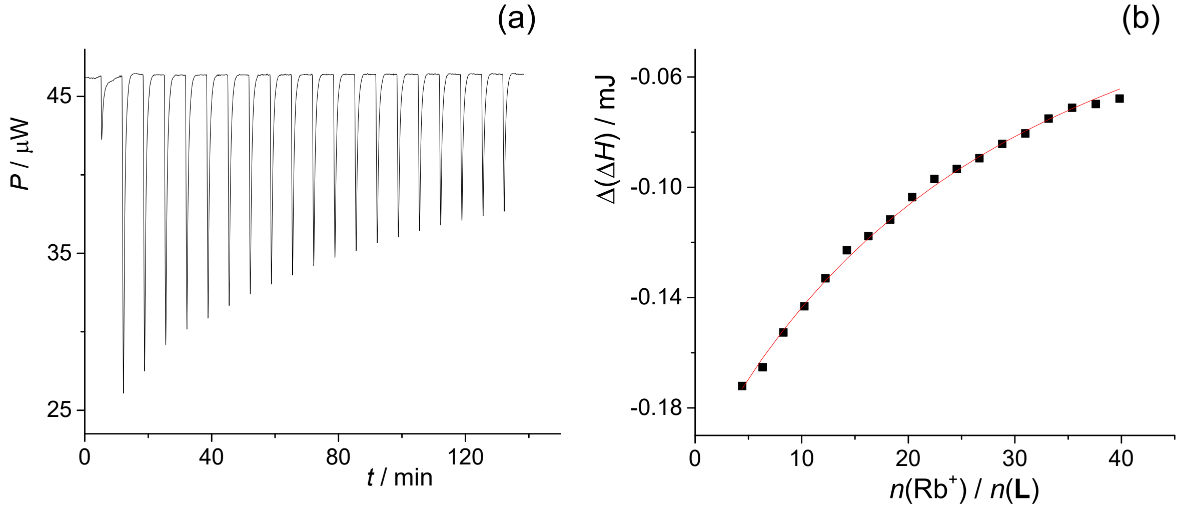

Figure S3. a) Microcalorimetric titration of **L** ($c = 1.24 \times 10^{-4}$ mol dm^{-3}, $V = 1.42$ mL) with RbCl ($c = 2.20 \times 10^{-2}$ mol dm^{-3}) in methanol at 25 °C; b) Dependence of successive enthalpy change on n(RbCl) / n(**L**) ratio. ■ experimental; — calculated.

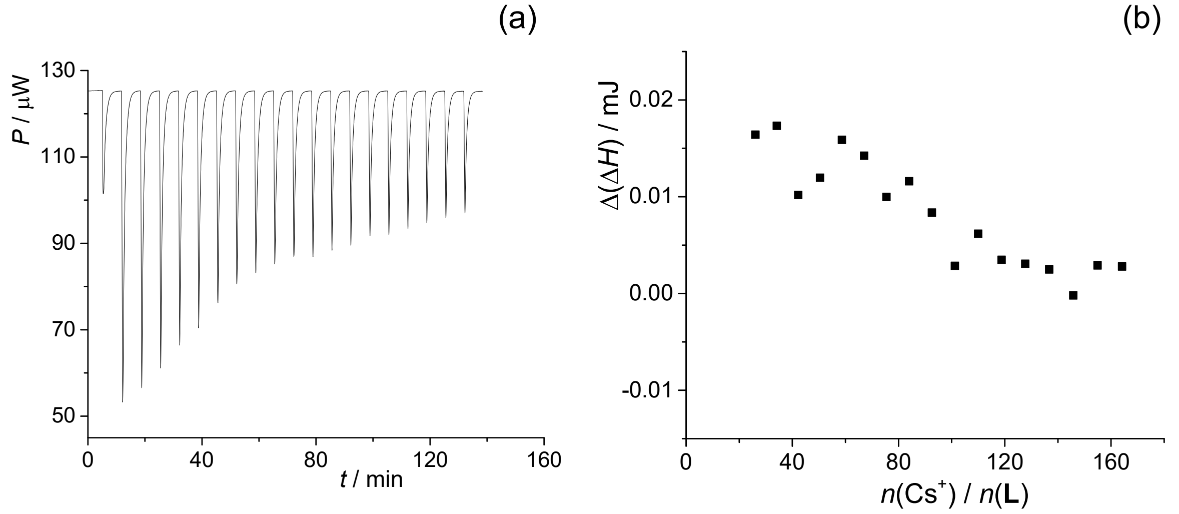

Figure S4. a) Microcalorimetric titration of **L** ($c = 1.23 \times 10^{-4}$ mol dm^{-3}, $V = 1.43$ mL) with CsCl ($c = 8.99 \times 10^{-2}$ mol dm^{-3}) in methanol at 25 °C; b) Dependence of successive enthalpy change on n(CsCl) / n(**L**) ratio.

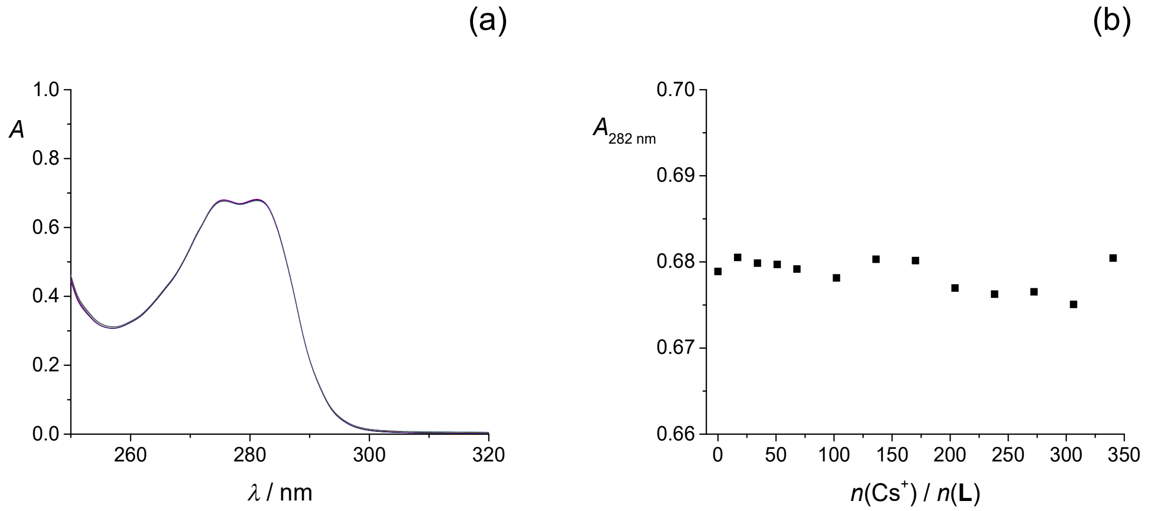

Figure S5. a) Spectrophotometric titration of **L** (c = 2.07 × 10^{-4} mol dm^{-3}, V_0 = 2.2 mL) with CsCl (c = 1.55 × 10^{-1} mol dm^{-3}) in methanol. l = 1 cm; ϑ = (25.0 ± 0.1) °C. The spectra are corrected for dilution. b) Dependence of absorbance at 282 nm on n(CsCl) / n(**L**) ratio. ■ experimental; — calculated.

2. Complexation of alkali metal cations with compound L in acetonitrile

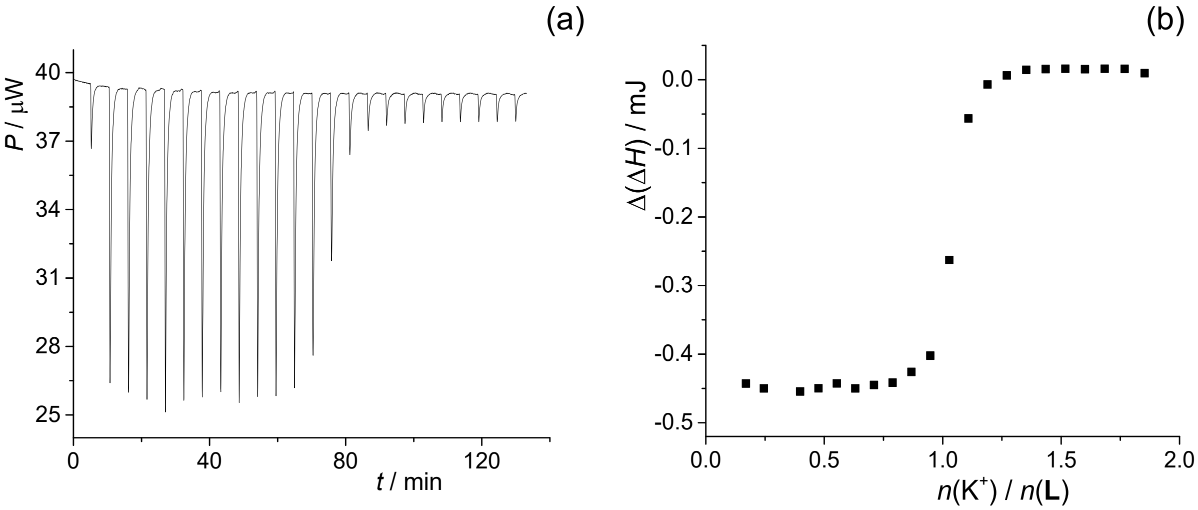

Figure S6. a) Microcalorimetric titration of **L** (c = 7.54 × 10^{-5} mol dm^{-3}, V = 1.42 mL) with $KClO_4$ (c = 1.00 × 10^{-3} mol dm^{-3}) in acetonitrile at 25 °C; b) Dependence of successive enthalpy change on $n(KClO_4)$ / n(**L**) ratio.

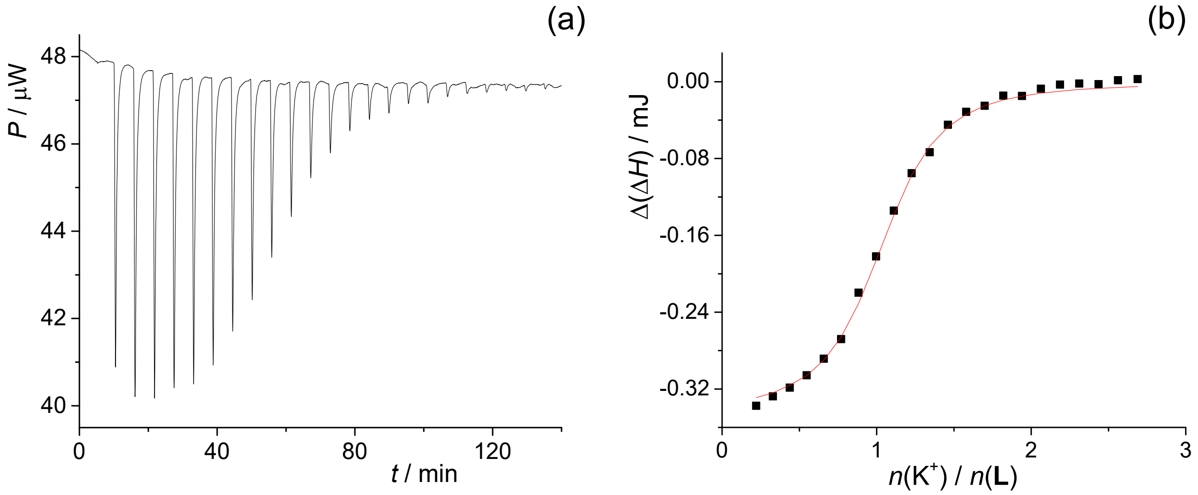

Figure S7. a) Microcalorimetric titration of Rb**L**$^+$ (c(**L**) = 1.73 × 10^{-4} mol dm^{-3}, c(RbI) = 2.58 × 10^{-3} mol dm^{-3}, V = 1.43 mL) with $KClO_4$ (c = 2.00 × 10^{-3} mol dm^{-3}) in acetonitrile at 25 °C; b) Dependence of successive enthalpy change on $n(KClO_4)$ / n(**L**) ratio. ■ experimental; — calculated.

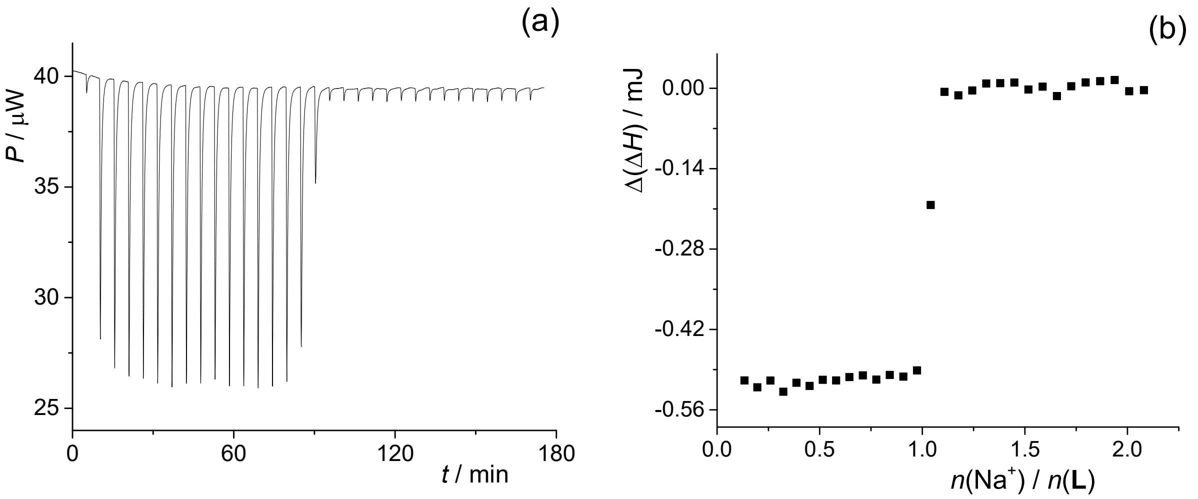

Figure S8. a) Microcalorimetric titration of **L** (c = 7.85 × 10^{-5} mol dm^{-3}, V = 1.43 mL) with $NaClO_4$ (c = 9.93 × 10^{-4} mol dm^{-3}) in acetonitrile at 25 °C; b) Dependence of successive enthalpy change on $n(NaClO_4)$ / n(**L**) ratio.

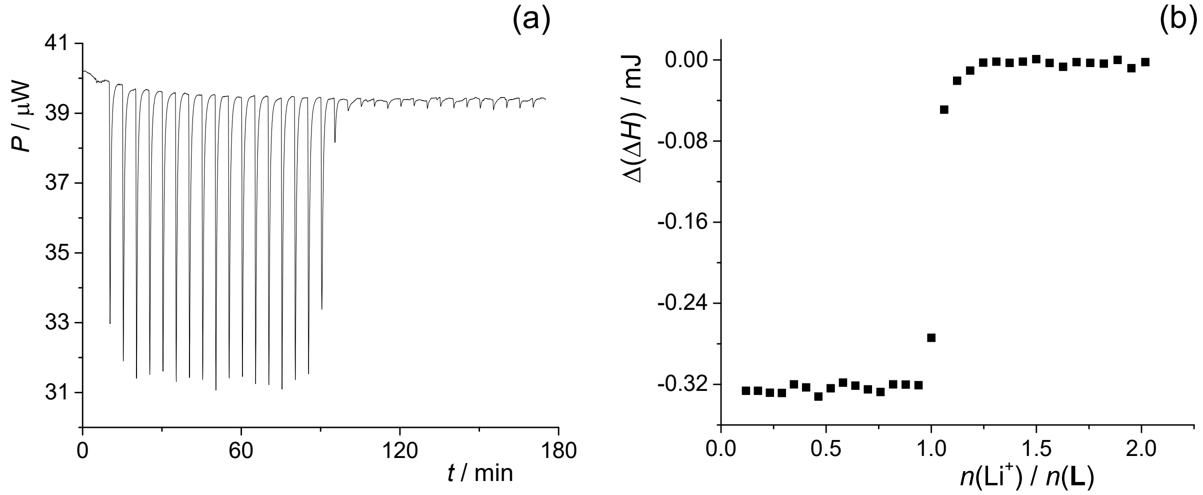

Figure S9. a) Microcalorimetric titration of **L** (c = 8.32 × 10^{-5} mol dm^{-3}, V = 1.43 mL) with $LiClO_4$ (c = 8.27 × 10^{-4} mol dm^{-3}) in acetonitrile at 25 °C; b) Dependence of successive enthalpy change on $n(LiClO_4)$ / n(**L**) ratio.

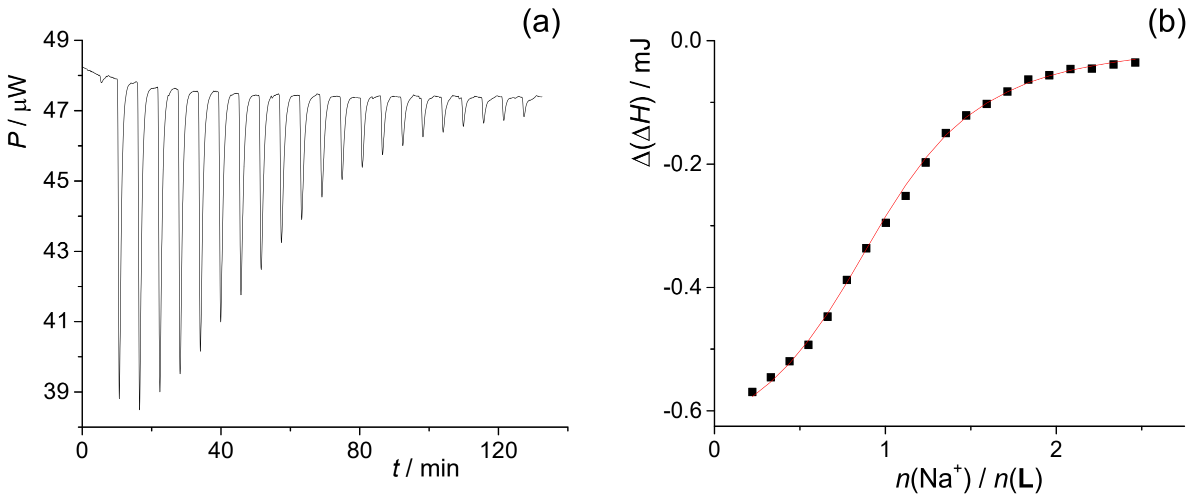

Figure S10. a) Microcalorimetric titration of LiL^+ (c(**L**) = 1.87 × 10^{-4} mol dm^{-3}, $c(LiClO_4)$ = 4.14 × 10^{-4} mol dm^{-3}, V = 1.43 mL) with $NaClO_4$ (c = 2.02 × 10^{-3} mol dm^{-3}) in acetonitrile at 25 °C; b) Dependence of successive enthalpy change on $n(NaClO_4)$ / n(**L**) ratio. ■ experimental; — calculated.

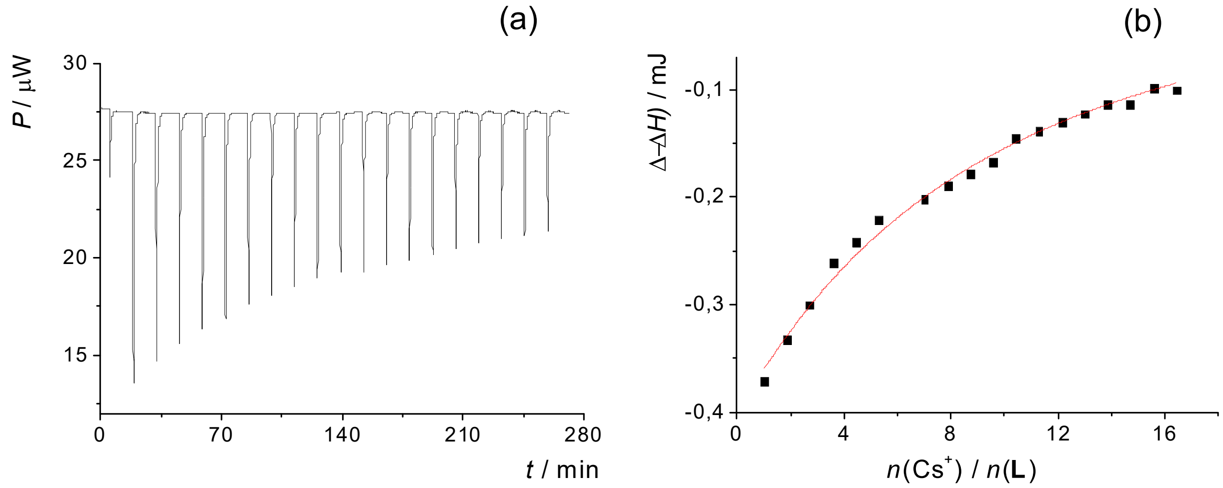

Figure S11. a) Microcalorimetric titration of **L** (c = 2.03 × 10^{-4} mol dm^{-3}, V = 1.45 mL) with CsI (c = 1.69 × 10^{-2} mol dm^{-3}) in acetonitrile at 25 °C; b) Dependence of successive enthalpy change on n(CsI) / n(**L**) ratio. ■ experimental; — calculated.

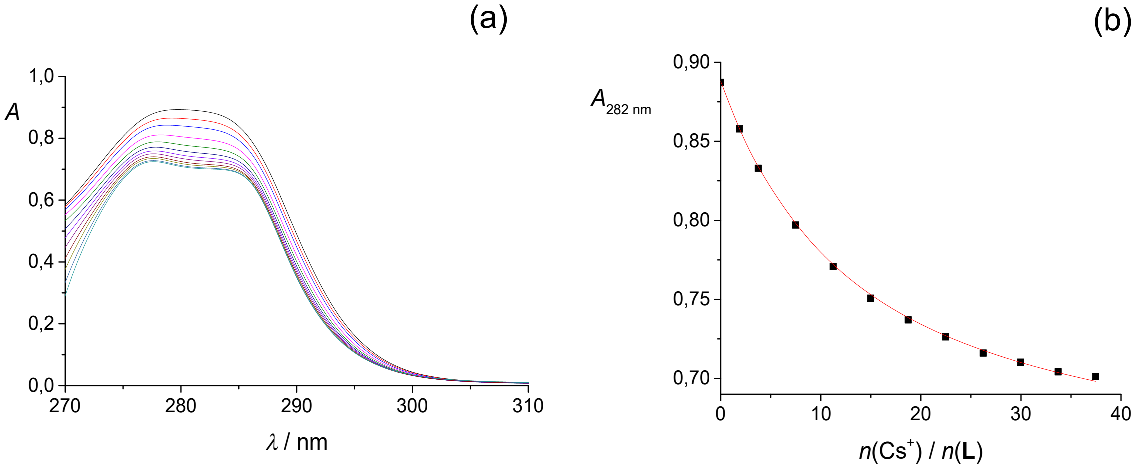

Figure S12. a) Spectrophotometric titration of **L** (c = 2.03 × 10^{-4} mol dm^{-3}, V_0 = 2.2 mL) with CsI (c = 1.67 × 10^{-2} mol dm^{-3}) in acetonitrile. l = 1 cm; ϑ = (25.0 ± 0.1) °C. The spectra are corrected for dilution. b) Dependence of absorbance at 282 nm on n(CsI) / n(**L**) ratio. ■ experimental; — calculated.

3. Complexation of alkali metal cations with compound L in *N,N*-dimethylformamide

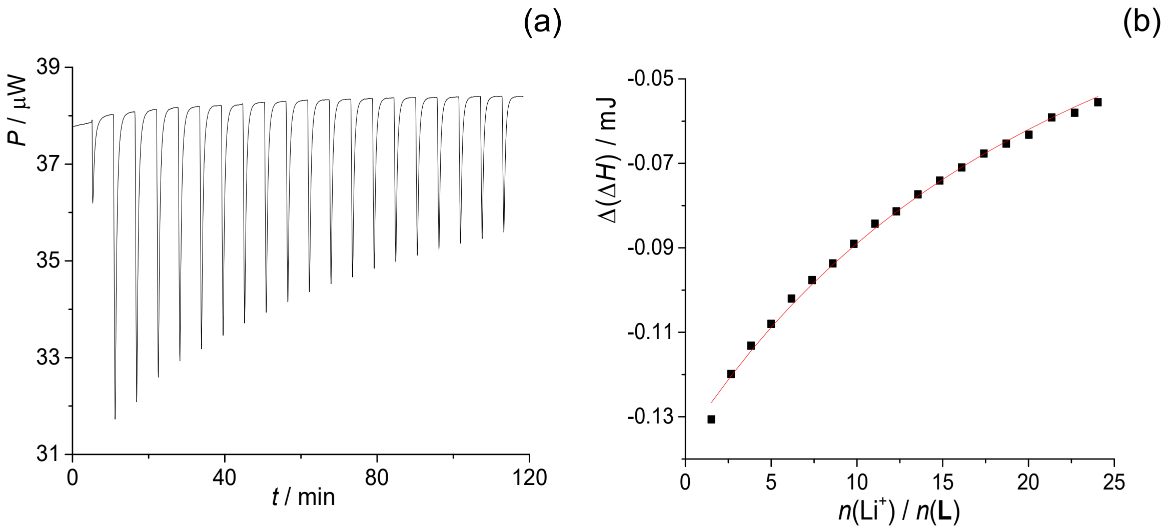

Figure S13. a) Microcalorimetric titration of **L** (c = 1.90 × 10^{-4} mol dm^{-3}, V = 1.42 mL) with $LiClO_4$ (c = 2.03 × 10^{-2} mol dm^{-3}) in *N,N*-dimethylformamide at 25 °C; b) Dependence of successive enthalpy change on $n(LiClO_4)$ / n(**L**) ratio. ■ experimental; — calculated.

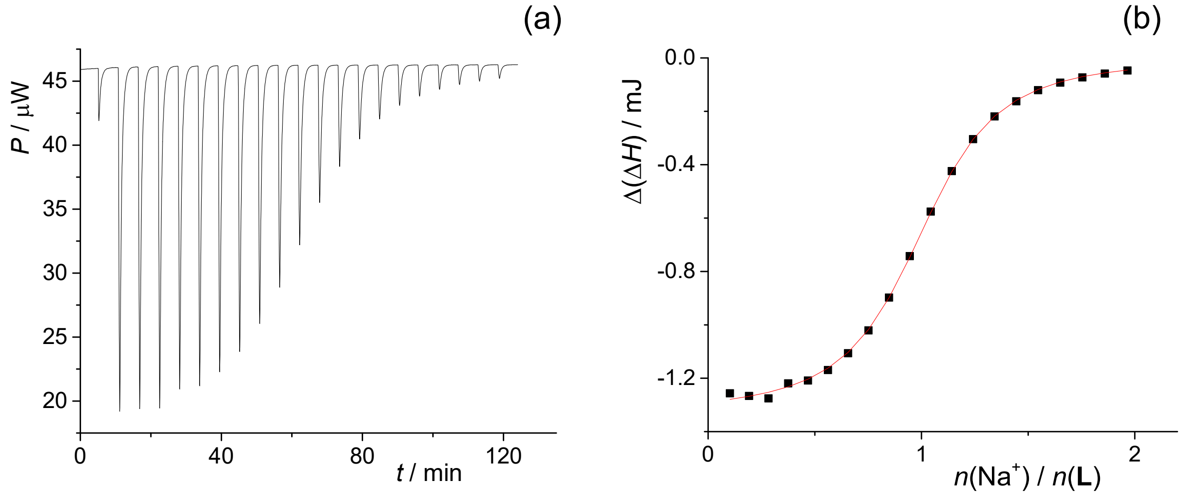

Figure S14. a) Microcalorimetric titration of **L** (c = 1.88 × 10^{-4} mol dm^{-3}, V = 1.42 mL) with $NaClO_4$ (c = 1.71 × 10^{-3} mol dm^{-3}) in *N,N*-dimethylformamide at 25 °C; b) Dependence of successive enthalpy change on $n(NaClO_4)$ / n(**L**) ratio. ■ experimental; — calculated.

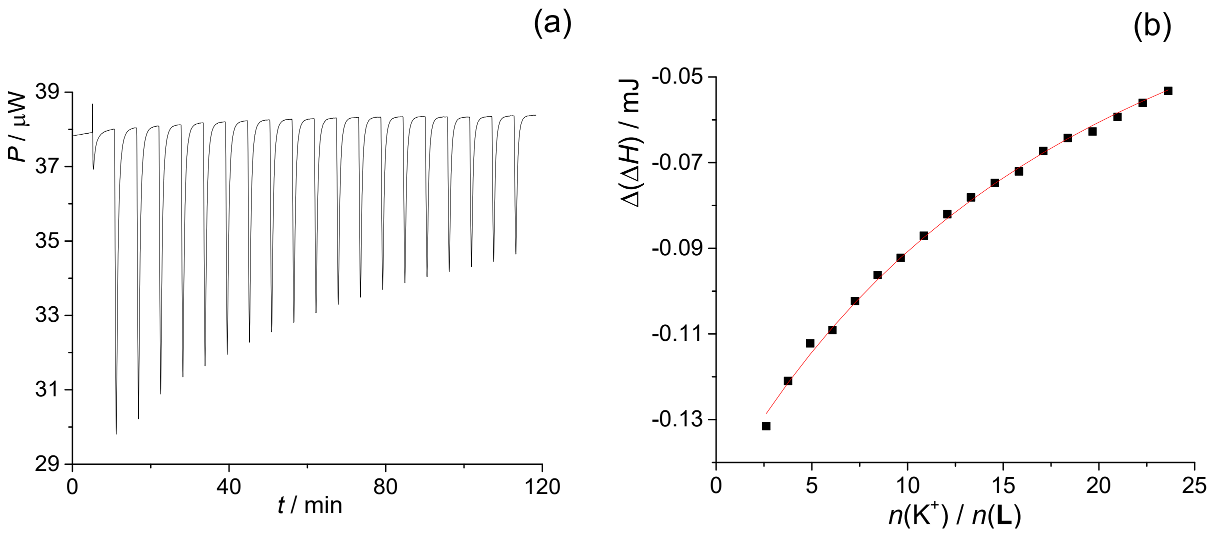

Figure S15. a) Microcalorimetric titration of **L** (c = 1.90 × 10^{-4} mol dm^{-3}, V = 1.42 mL) with $KClO_4$ (c = 1.99 × 10^{-2} mol dm^{-3}) in *N*,*N*-dimethylformamide at 25 °C; b) Dependence of successive enthalpy change on $n(KClO_4)$ / n(**L**) ratio. ■ experimental; — calculated.

4. Complexation of alkali metal cations with compound l in methanol

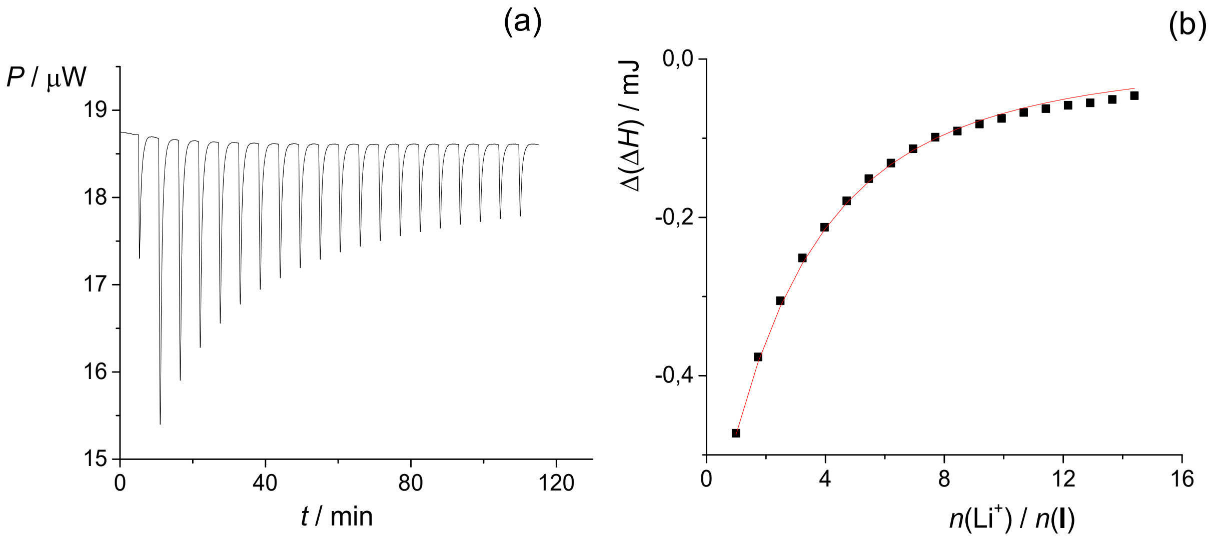

Figure S16. a) Microcalorimetric titration of **l** (c = 1.61 × 10^{-4} mol dm^{-3}, V = 1.42 mL) with $LiClO_4$ (c = 1.14 × 10^{-2} mol dm^{-3}) in methanol at 25 °C; b) Dependence of successive enthalpy change on $n(LiClO_4)$ / n(**l**) ratio. ■ experimental; — calculated.

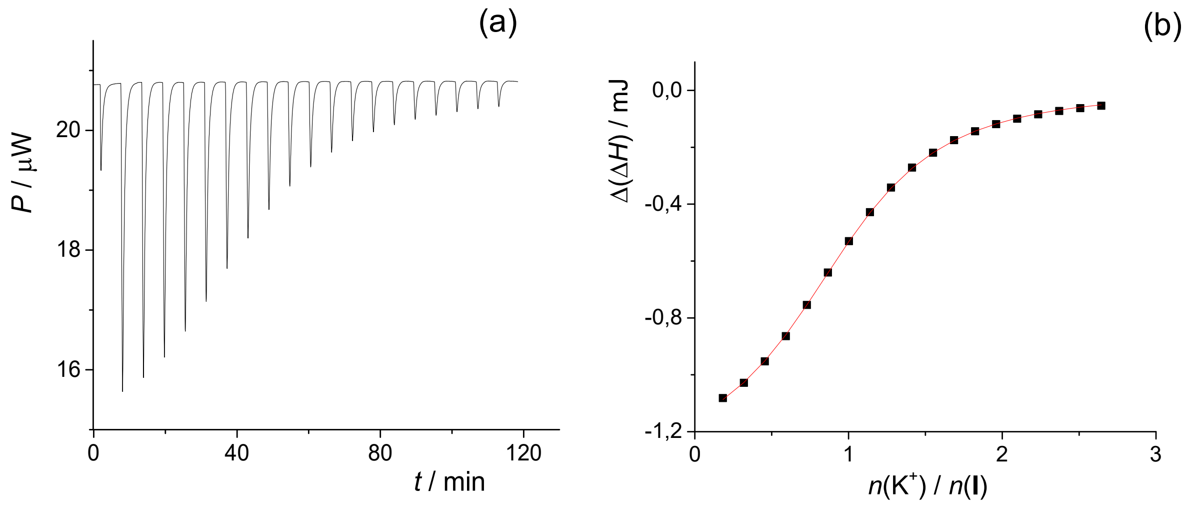

Figure S17. a) Microcalorimetric titration of **l** (c = 1.51 × 10^{-4} mol dm^{-3}, V = 1.42 mL) with $KClO_4$ (c = 1.96 × 10^{-3} mol dm^{-3}) in methanol at 25 °C; b) Dependence of successive enthalpy change on $n(KClO_4)$ / n(**l**) ratio. ■ experimental; — calculated.

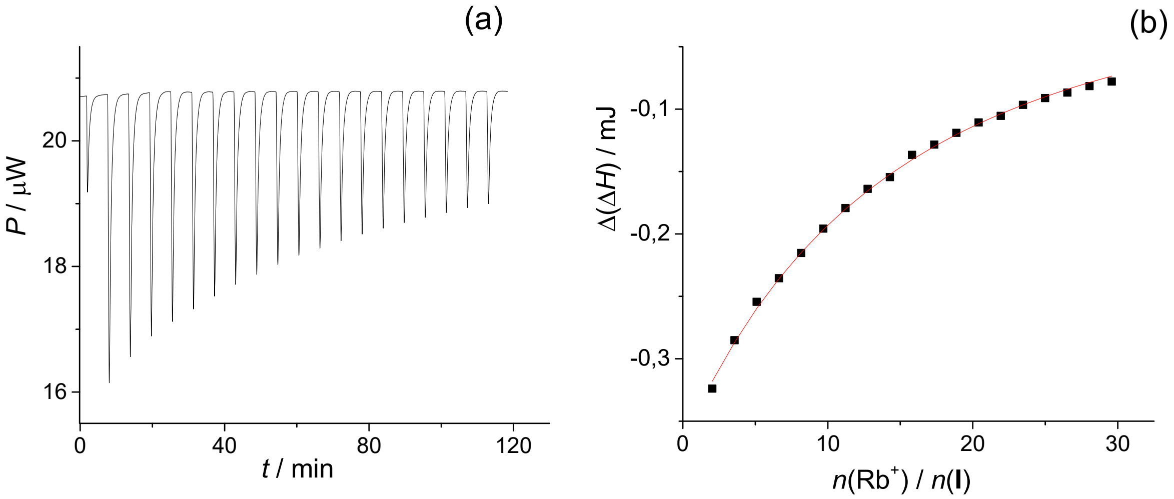

Figure S18. a) Microcalorimetric titration of **I** ($c = 1.51 \times 10^{-4}$ mol dm^{-3}, $V = 1.42$ mL) with RbI ($c = 2.19 \times 10^{-2}$ mol dm^{-3}) in methanol at 25 °C; b) Dependence of successive enthalpy change on n(RbI) / n(**I**) ratio. ■ experimental; — calculated.

5. Complexation of alkali metal cations with compound **1** in *N,N*-dimethylformamide

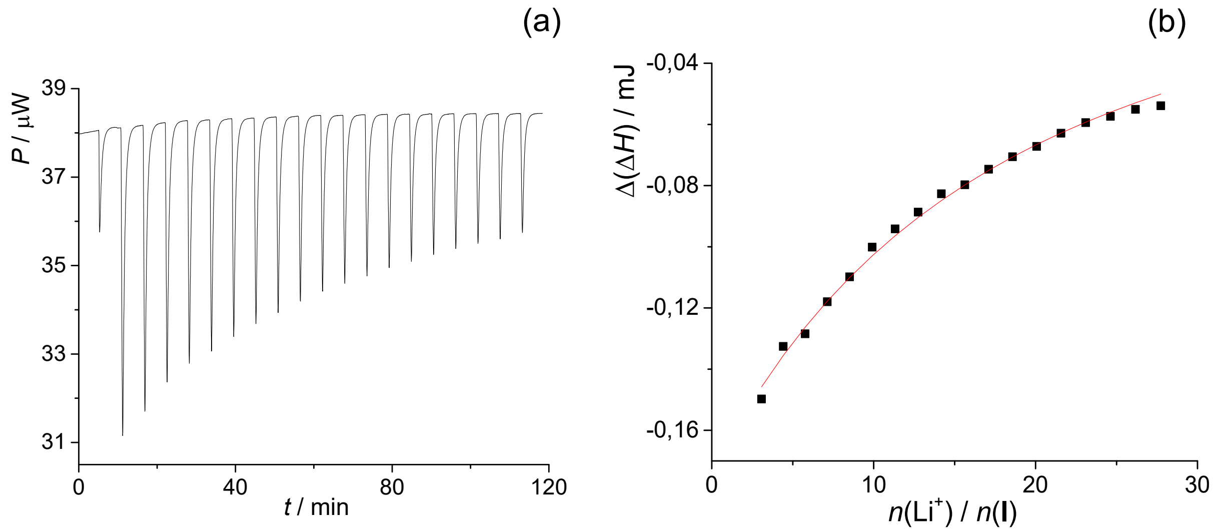

Figure S19. a) Microcalorimetric titration of **1** (c = 1.65 × 10^{-4} mol dm^{-3}, V = 1.42 mL) with $LiClO_4$ (c = 2.03 × 10^{-2} mol dm^{-3}) in *N,N*-dimethylformamide at 25 °C; b) Dependence of successive enthalpy change on $n(LiClO_4)$ / n(**1**) ratio. ■ experimental; — calculated.

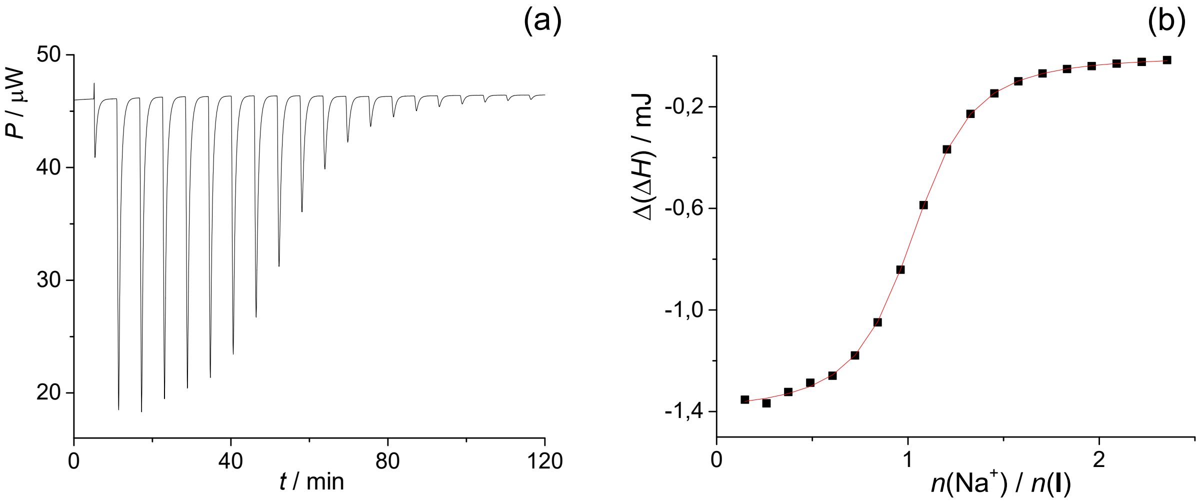

Figure S20. a) Microcalorimetric titration of **1** (c = 1.64 × 10^{-4} mol dm^{-3}, V = 1.42 mL) with $NaClO_4$ (c = 1.71 × 10^{-3} mol dm^{-3}) in *N,N*-dimethylformamide at 25 °C; b) Dependence of successive enthalpy change on $n(NaClO_4)$ / n(**1**) ratio. ■ experimental; — calculated.

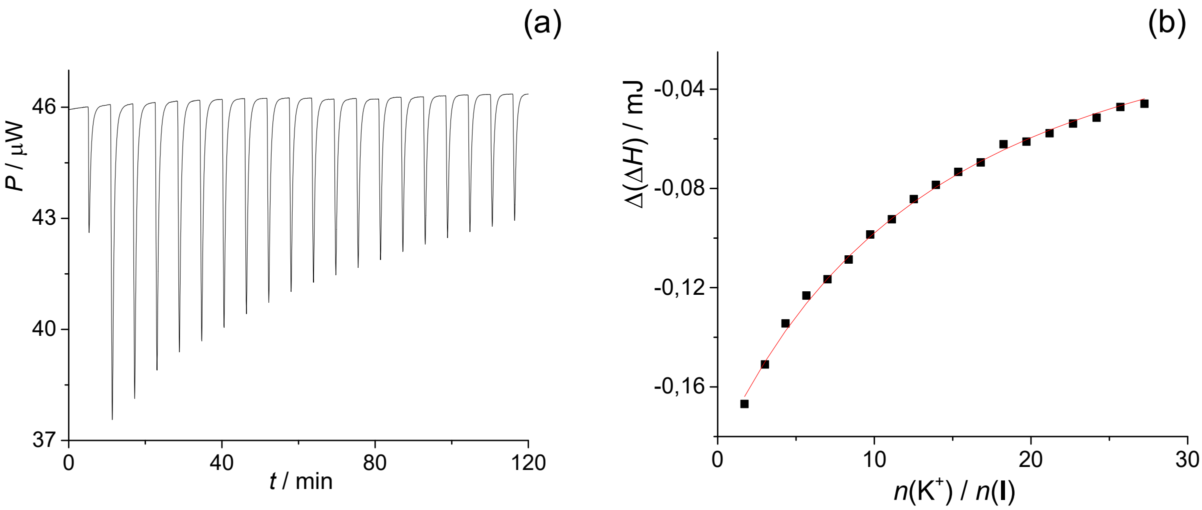

Figure S21. a) Microcalorimetric titration of **l** (c = 1.65 × 10^{-4} mol dm^{-3}, V = 1.42 mL) with $KClO_4$ (c = 1.99 × 10^{-3} mol dm^{-3}) in *N*,*N*-dimethylformamide at 25 °C; b) Dependence of successive enthalpy change on $n(KClO_4)$ / n(**l**) ratio. ■ experimental; — calculated.

Table S1. The differences in standard thermodynamic parameters for complexation of alkali metal cation reactions with receptors **L** and **l** in examined solvents at 25 °C. ($\Delta(\Delta_r X°) = \Delta_r X°(ML^+) - \Delta_r X°(ML^+)$; $X \in \{G, H, S\}$).

**solvent**	**cation**	$\Delta(\Delta_r G°)$ / kJ mol^{-1}	$\Delta(\Delta_r H°)$ / kJ mol^{-1}	$\Delta(T\Delta_r S°)$ / kJ mol^{-1}
MeOH	Li^+	1.6	1.3	–0.3
	Na^+	–0.7	–1.9	–1.2
	K^+	0.4	0.3	–0.1
	Rb^+	1.7	–8.2	–9.9
DMF	Li^+	1.5	–5.7	–7.2
	Na^+	1.2	–0.9	–2.1
	K^+	1.8	–7.2	–9.0

6. The solvation of receptors in studied solvents

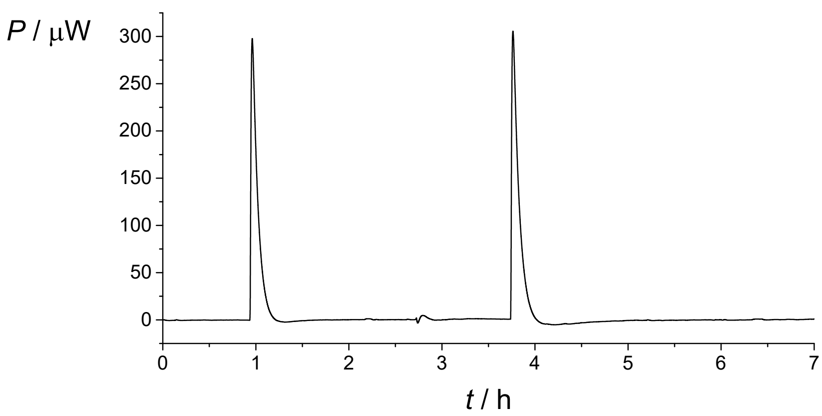

Figure S22. Dissolution of **l** (m_1 = 5.69 mg, m_2 = 6.52 mg) in MeOH (V = 17 mL) at 25 °C.

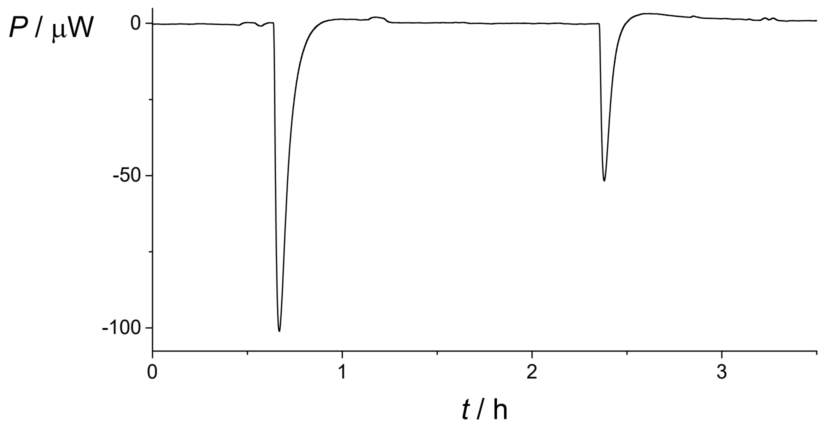

Figure S23. Dissolution of **L** (m_1 = 5.97 mg, m_2 = 3.09 mg) in MeOH (V = 17 mL) at 25 °C.

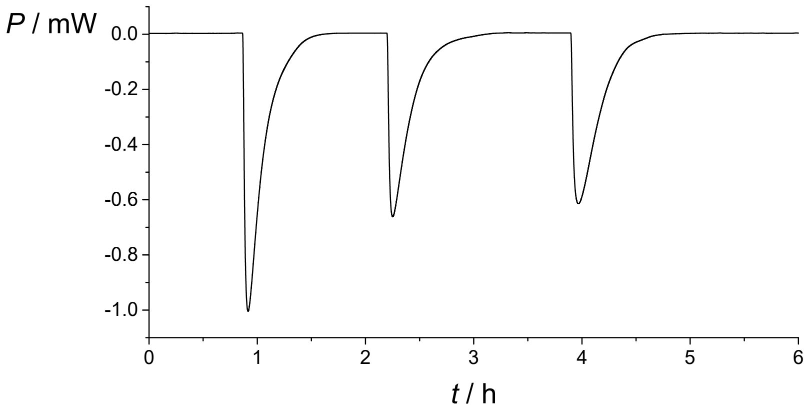

Figure S24. Dissolution of **Glc** (m_1 = 7.37 mg, m_2 = 5.57 mg, m_3 = 6.28 mg) in MeOH (V = 17 mL) at 25 °C.

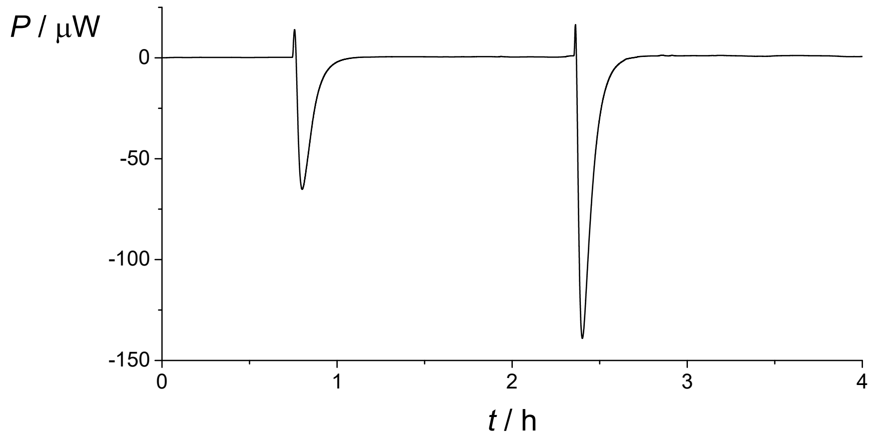

Figure S25. Dissolution of **L** (m_1 = 3.04 mg, m_2 = 5.04 mg) in DMF (V = 17 mL) at 25 °C.

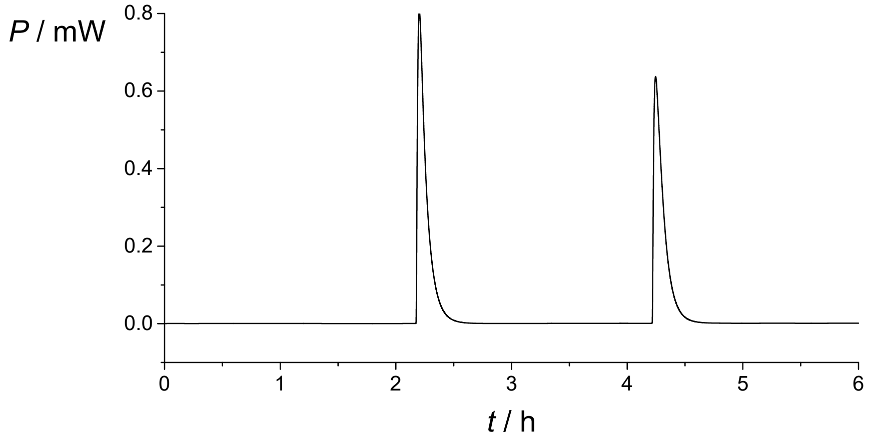

Figure S26. Dissolution of **l** (m_1 = 6.14 mg, m_2 = 5.60 mg) in DMF (V = 17 mL) at 25 °C.

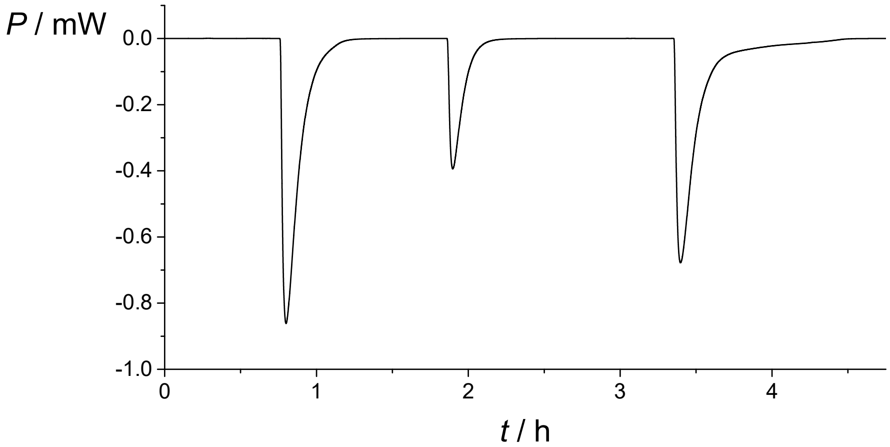

Figure S27. Dissolution of **Glc** (m_1 = 8.30 mg, m_2 = 3.00 mg, m_3 = 8.42 mg) in DMF (V = 17 mL) at 25 °C.

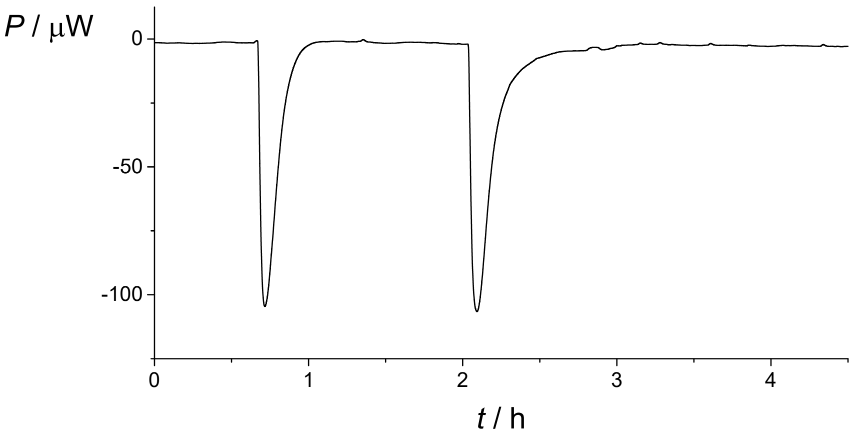

Figure S28. Dissolution of **L** (m_1 = 3.02 mg, m_2 = 4.15 mg) in MeCN (V = 17 mL) at 25 °C.

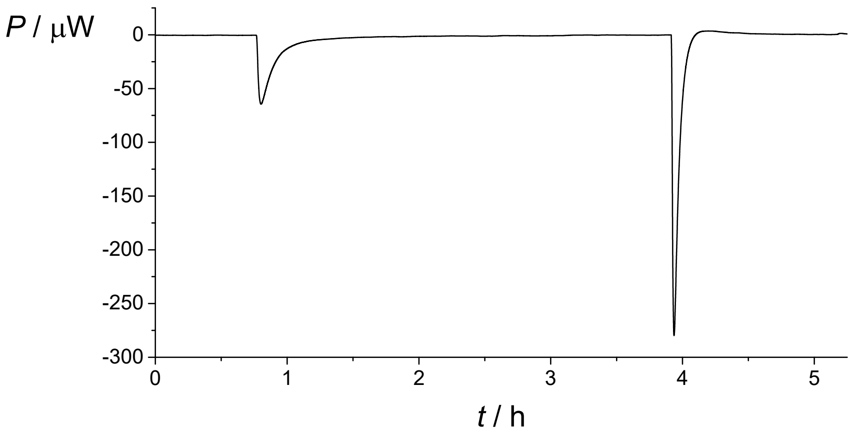

Figure S29. Dissolution of **l** (m_1 = 1.55 mg, m_2 = 1.45 mg) in MeCN (V = 17 mL) at 25 °C.

7. Inclusion of solvent molecule into calixarene L

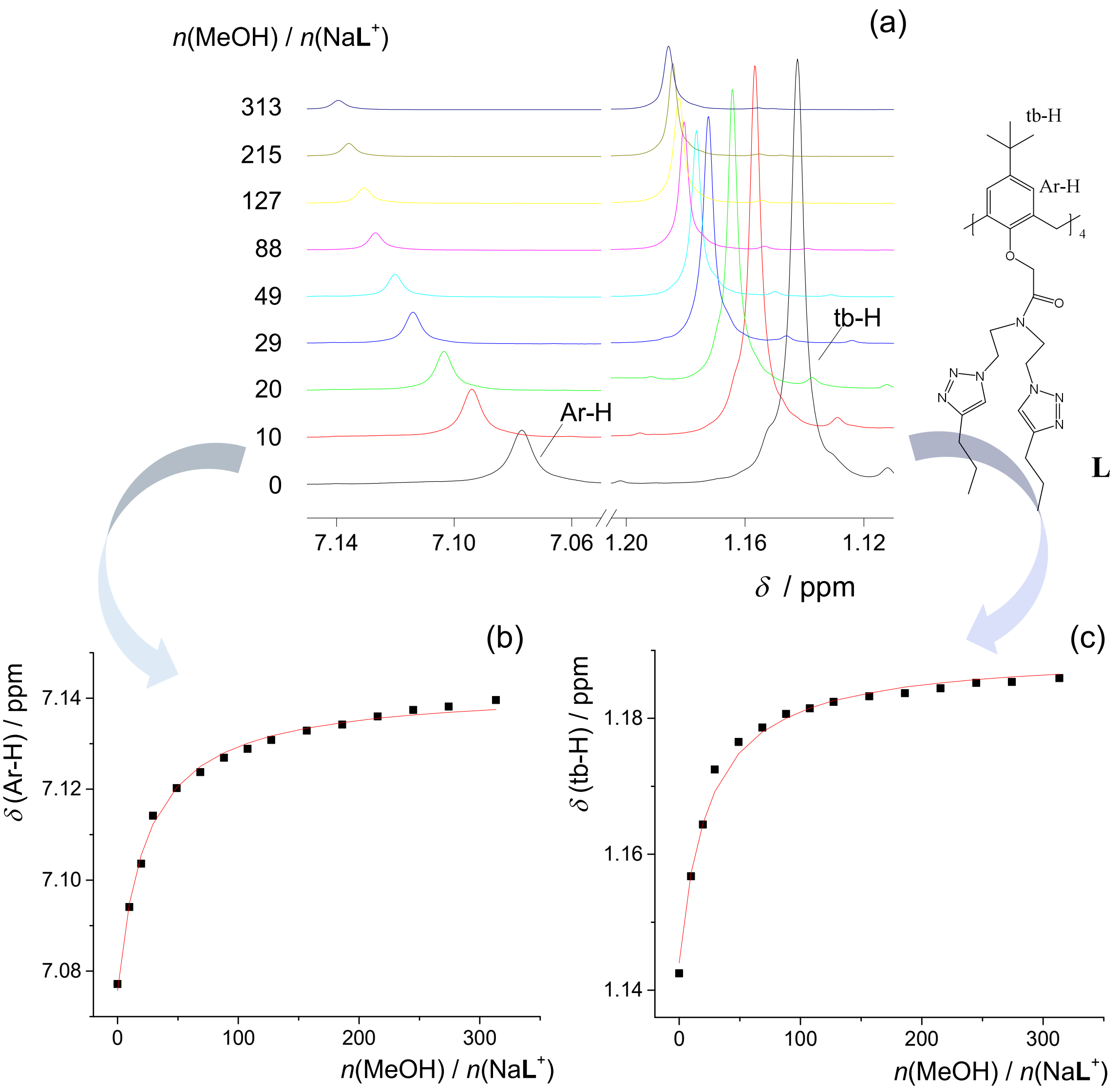

Figure S30. a) ^{1}H NMR spectroscopy titration of Na**L**ClO_4 (c = 6.02 × 10^{-3} mol dm^{-3}, V_0 = 500 µL) with MeOH (c = 5.892 mol dm^{-3}) in $CDCl_3$ at 25 °C. Selected ^{1}H NMR spectra acquired during the titration; augmented parts of the spectra that exhibit most important information are shown. b), c) Experimental (■) and calculated (—) chemical shifts.

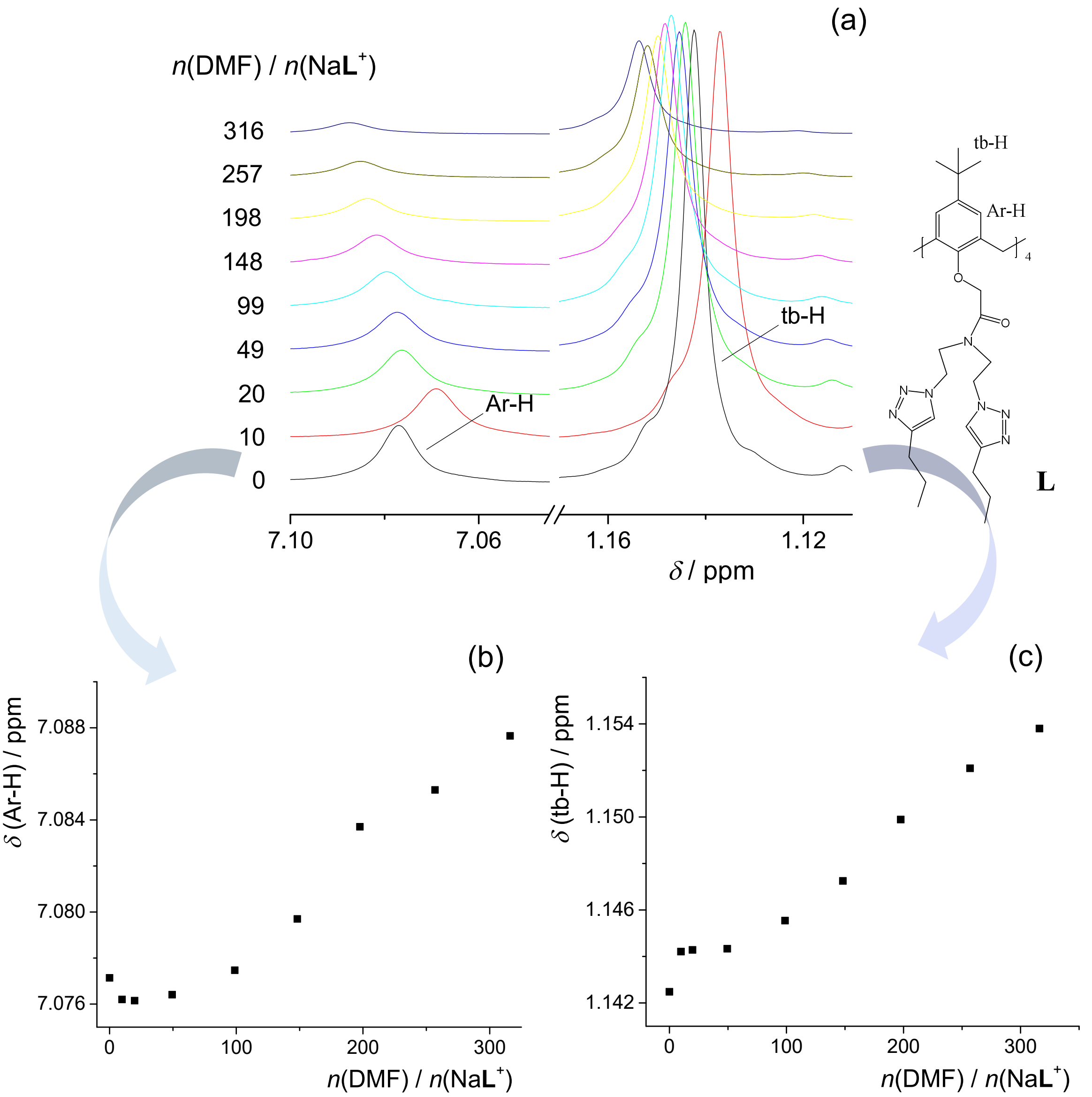

Figure S31. a) ^{1}H NMR spectroscopy titration of Na**L**ClO$_4$ (c = 6.02 × 10^{-3} mol dm^{-3}, V_0 = 500 μL) with DMF (c = 5.892 mol dm^{-3}) in CDCl$_3$ at 25 °C. Selected ^{1}H NMR spectra acquired during the titration; augmented parts of the spectra that exhibit most important information are shown. b), c) Experimental chemical shifts.

8. **The solvent effect on the alkali metal complexation and comparison of L and l binding affinities**

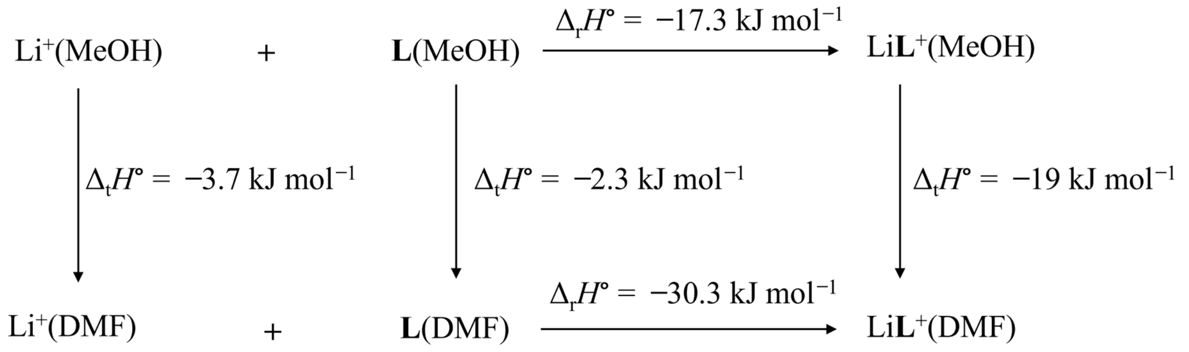

Scheme S1. Thermodynamic cycle explaining the differences in standard complexation enthalpies of lithium cation with compound **L** in MeOH and DMF.

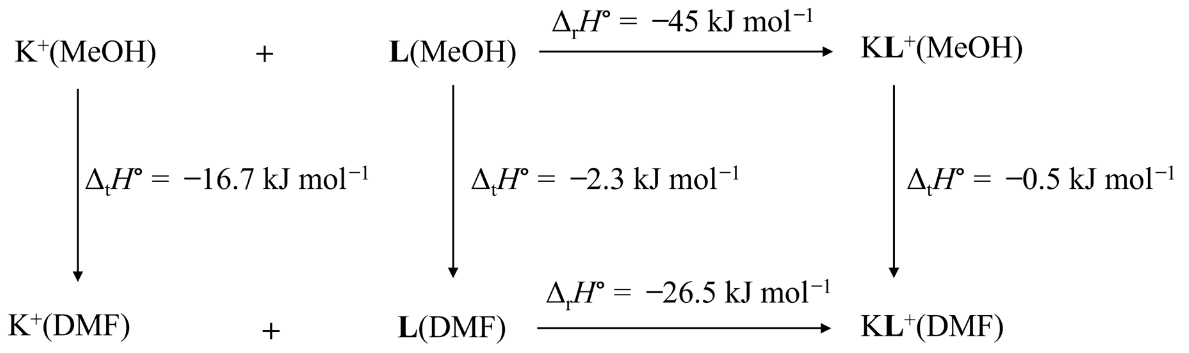

Scheme S2. Thermodynamic cycle explaining the differences in standard complexation enthalpies of potassium cation with compound **L** in MeOH and DMF.

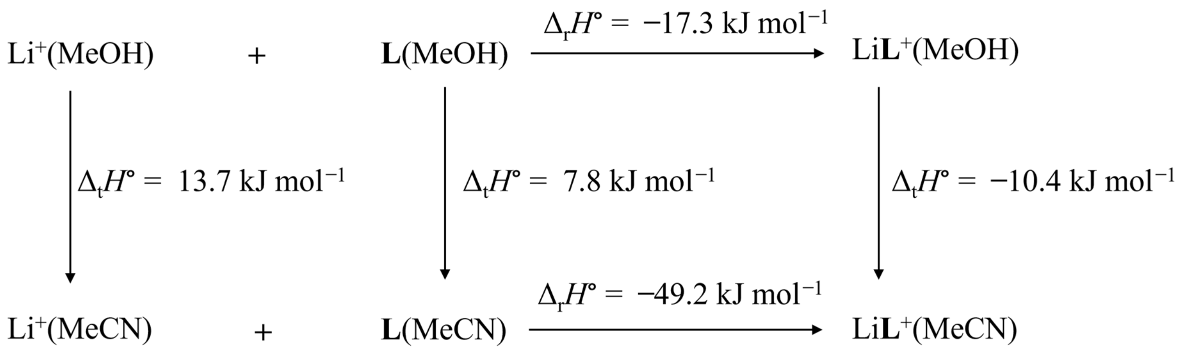

Scheme S3. Thermodynamic cycle explaining the differences in standard complexation enthalpies of lithium cation with compound **L** in MeOH and MeCN.

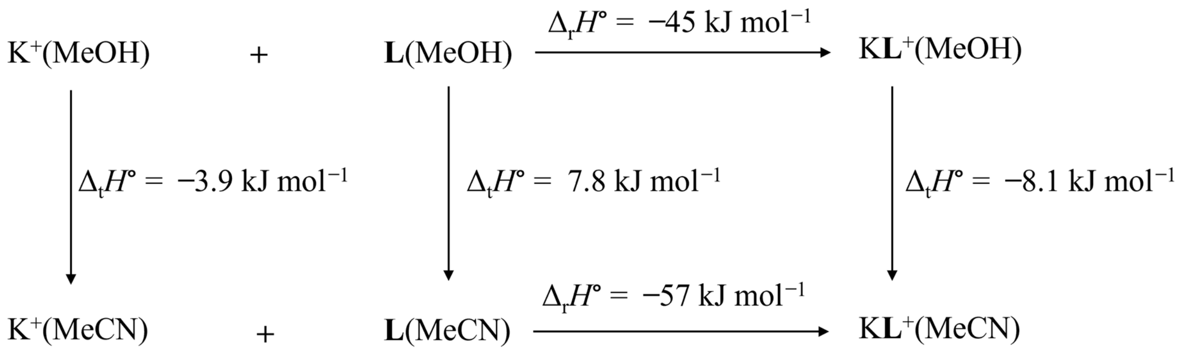

Scheme S4. Thermodynamic cycle explaining the differences in standard complexation enthalpies of potassium cation with compound **L** in MeOH and MeCN.

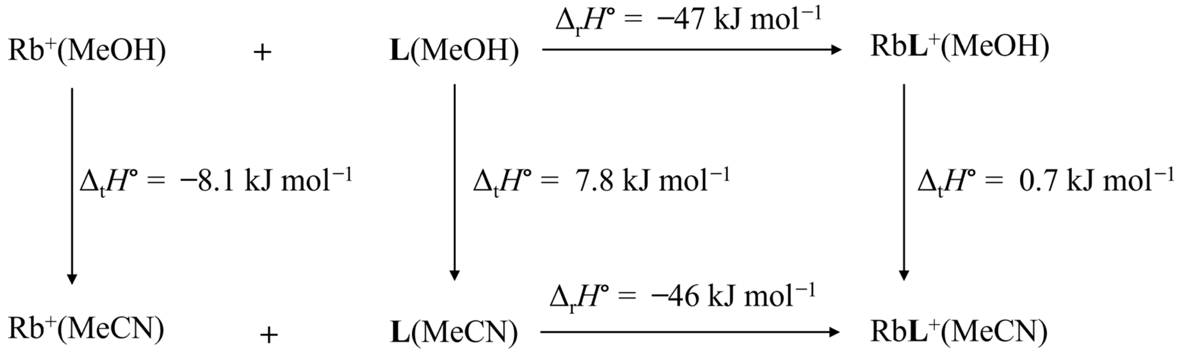

Scheme S5. Thermodynamic cycle explaining the differences in standard complexation enthalpies of rubidium cation with compound **L** in MeOH and MeCN.

$$Li^+(MeOH) + \mathbf{l}(MeOH) \xrightarrow{\Delta_r H^\circ = -19\ kJ\ mol^{-1}} Li\mathbf{l}^+(MeOH)$$

$Li^+(MeOH) \rightarrow Li^+(DMF)$: $\Delta_t H^\circ = -3.7$ kJ mol^{-1}

$\mathbf{l}(MeOH) \rightarrow \mathbf{l}(DMF)$: $\Delta_t H^\circ = -83.3$ kJ mol^{-1}

$Li\mathbf{l}^+(MeOH) \rightarrow Li\mathbf{l}^+(DMF)$: $\Delta_t H^\circ = -92.6$ kJ mol^{-1}

$$Li^+(DMF) + \mathbf{l}(DMF) \xrightarrow{\Delta_r H^\circ = -24.6\ kJ\ mol^{-1}} Li\mathbf{l}^+(DMF)$$

Scheme S6. Thermodynamic cycle explaining the differences in standard complexation enthalpies of lithium cation with compound **l** in MeOH and DMF.

$$K^+(MeOH) + \mathbf{l}(MeOH) \xrightarrow{\Delta_r H^\circ = -45.3\ kJ\ mol^{-1}} K\mathbf{l}^+(MeOH)$$

$K^+(MeOH) \rightarrow K^+(DMF)$: $\Delta_t H^\circ = -16.7$ kJ mol^{-1}

$\mathbf{l}(MeOH) \rightarrow \mathbf{l}(DMF)$: $\Delta_t H^\circ = -83.3$ kJ mol^{-1}

$K\mathbf{l}^+(MeOH) \rightarrow K\mathbf{l}^+(DMF)$: $\Delta_t H^\circ = -74$ kJ mol^{-1}

$$K^+(DMF) + \mathbf{l}(DMF) \xrightarrow{\Delta_r H^\circ = -19.3\ kJ\ mol^{-1}} K\mathbf{l}^+(DMF)$$

Scheme S7. Thermodynamic cycle explaining the differences in standard complexation enthalpies of potassium cation with compound **l** in MeOH and DMF.

9. Molecular dynamics simulations: Structures of L and l in MeCN, MeOH, and DMF

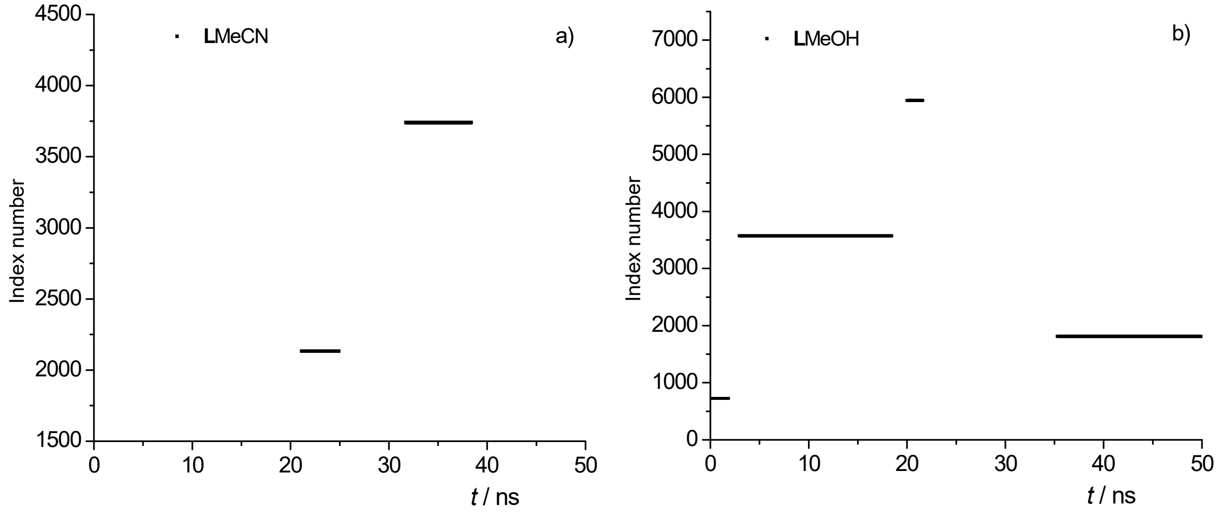

Figure S32. Index number of solvent molecules that occupy hydrophobic cavities of **L** during MD simulations in a) acetonitrile and b) methanol at 25 °C.

Table S2. Energies of interactions of **L** with solvent molecules, occurrence time ratio of different chemical species and average calixarene basket geometry obtained by MD simulations in examined solvents at 25 °C; d_{ref} = 7,85 Å.

	**MeCN**		**MeOH**		**DMF**
	**LMeCN**	**L**	**LMeOH**	**L**	**L**
E(**L**–Solvent) / kJ mol^{-1}	–1344	–1265	–1541	–1461	–1319
E(**L**–Solvent$_{incl}$) / kJ mol^{-1}	–50	–	–43	–	–
t_{total} / ns	50		50		50
t / t_{total}	0.21	0.79	0.67	0.33	1.000
N(Solvent$_{incl}$)	2	–	4	–	–
$\bar{d}$ / Å	7.56	5.81	7.72	6.16	5.67
	8.27	9.31	7.97	8.86	9.49
σ(d) / Å	0.27	0.35	0.29	0.60	0.28
	0.37	0.29	0.28	0.47	0.25
$\|d - d_{ref}\|$ / Å	0.33	2.04	0.25	1.73	2.28
	0.45	1.46	0.25	1.07	1.64

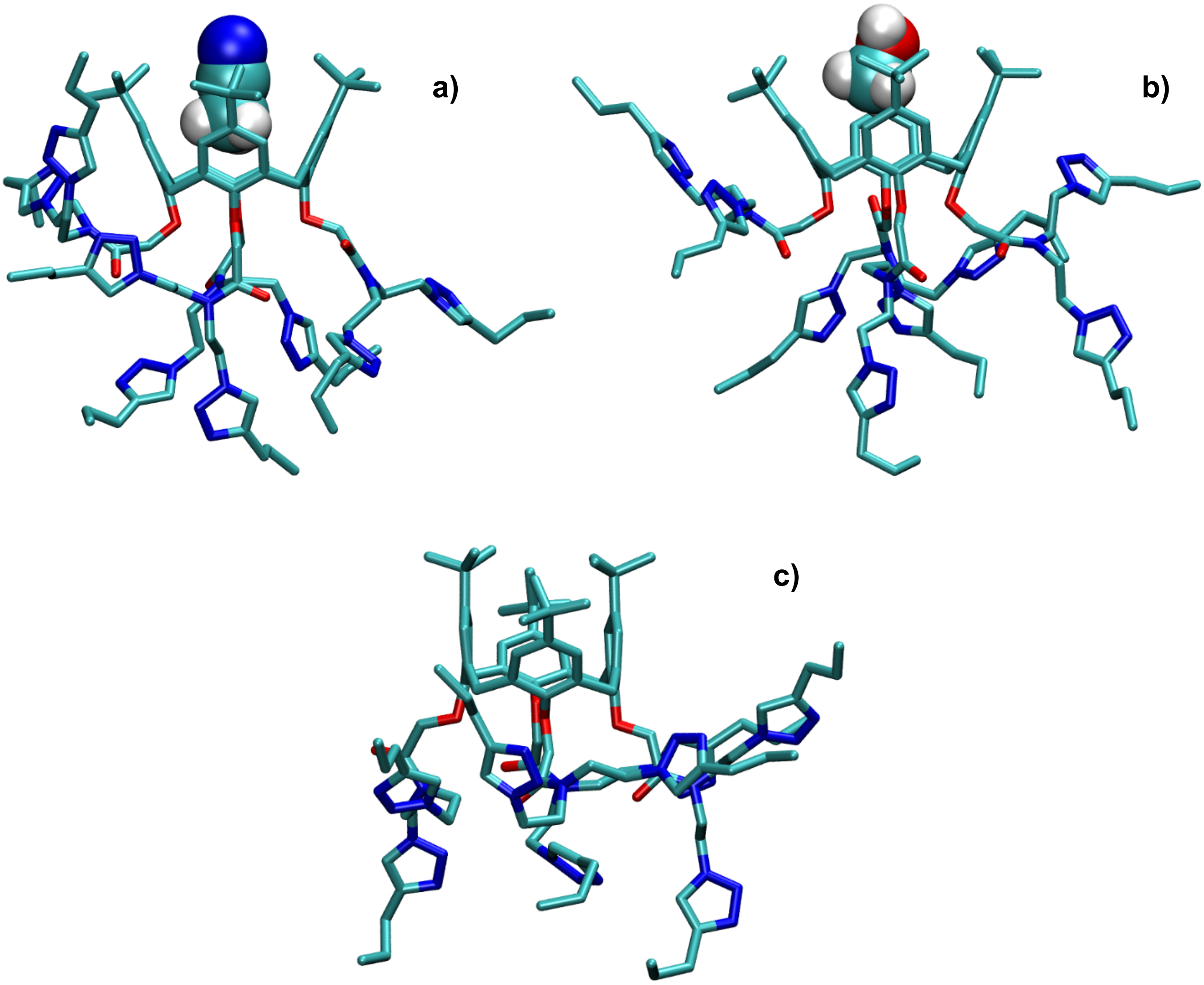

Figure S33. Structures of a) **L**MeCN, b) **L**MeOH, and c) free **L** in DMF obtained by MD simulations at 25 °C. Hydrogen atoms of **L** are omitted for clarity.

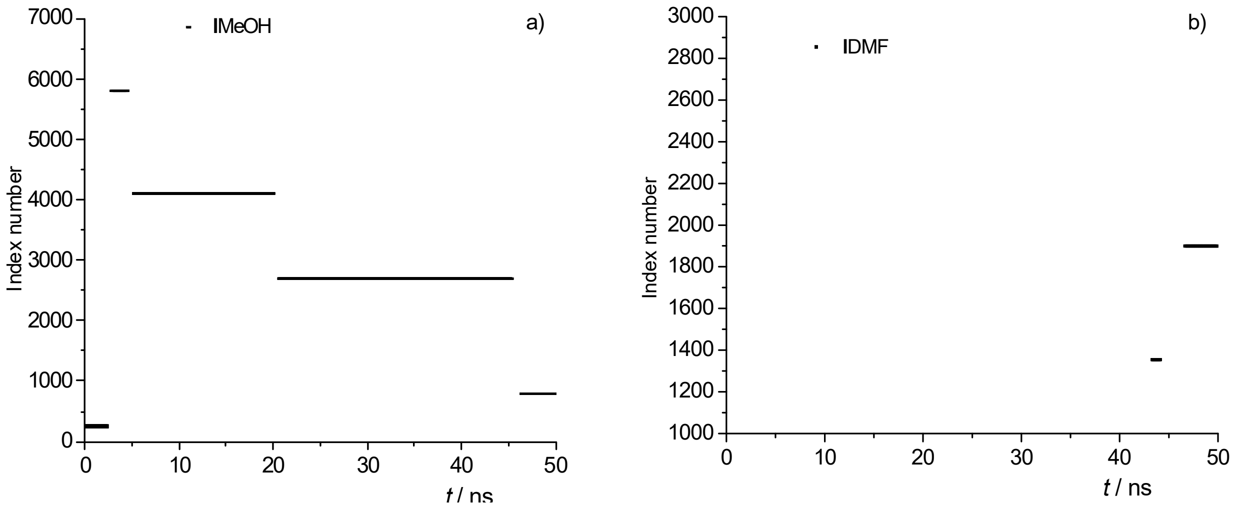

Figure S34. Index number of methanol and *N,N*-dimethylformamide molecules that occupy hydrophobic cavity of **1** during MD simulations in a) methanol and b) *N,N*-dimethylformamide at 25 °C.

Table S3. Energies of interactions of **1** with solvent molecules, occurrence time ratio of different chemical species and average calixarene basket geometry obtained by MD simulations in examined solvents at 25 °C; d_{ref} = 7,85 Å.

	**MeOH**		**DMF**	
	**1**MeOH	**1**	**1**DMF	**1**
E(**1**–Solvent) / kJ mol^{-1}	–2305	–2292	–2203	–2080
E(**1**–Solvent$_{incl}$) / kJ mol^{-1}	–44	–	–58	–
t_{total} / ns	50		50	
t / t_{total}	0.94	0.06	0.09	0.91
N(Solvent$_{incl}$)	5	–	2	–
$\bar{d}$ / Å	7.78	7.27	7.93	6.55
	7.90	8.07	8.07	8.55
σ(d) / Å	0.26	0.60	0.31	0.47
	0.26	0.49	0.31	0.35
$\lvert d - d_{ref} \rvert$ / Å	0.22	0.68	0.26	1.30
	0.21	0.44	0.31	0.72

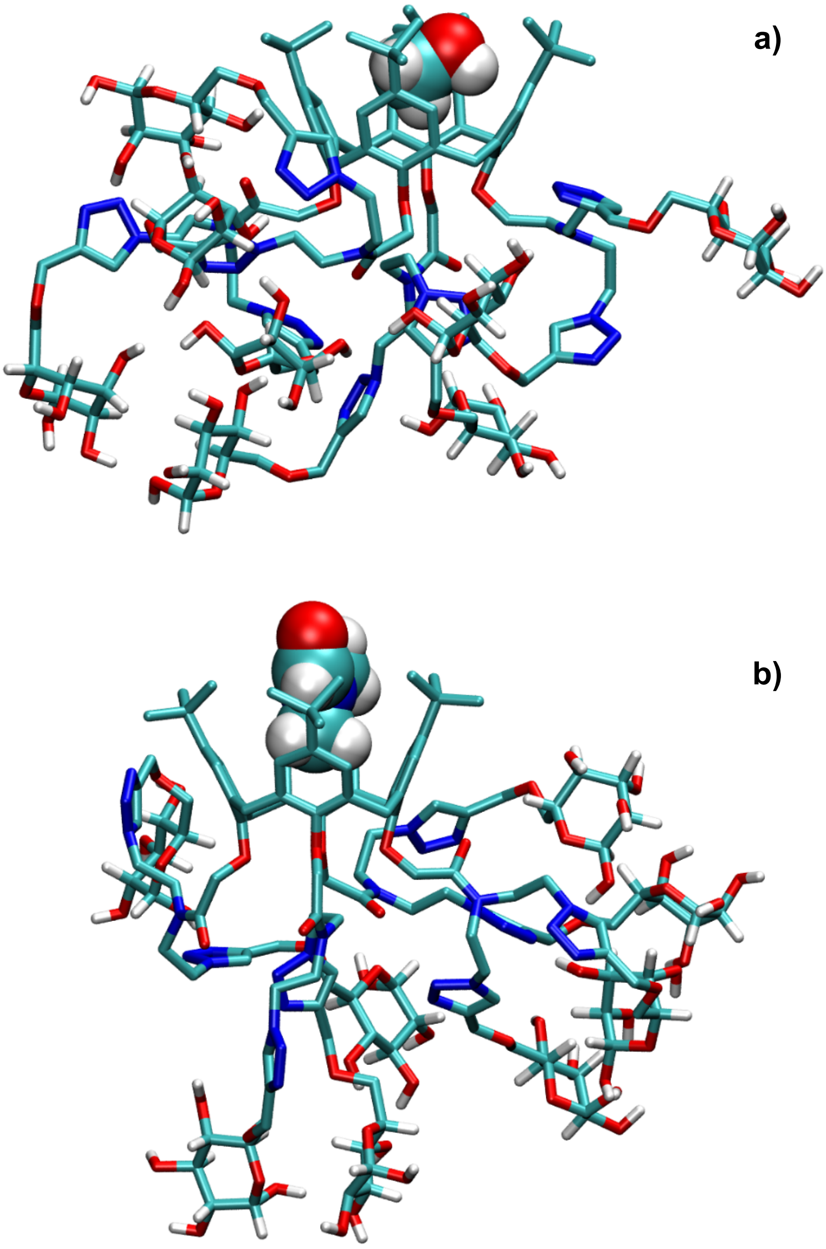

Figure S35. Structures of a) **1**MeOH and b) **1**DMF obtained by MD simulations in methanol and *N,N*-dimethylformamide at 25 °C. Non-glucose hydrogen atoms of **1** are omitted for clarity.

10. Molecular dynamics simulations: Complexes of L and l in MeCN, MeOH and DMF

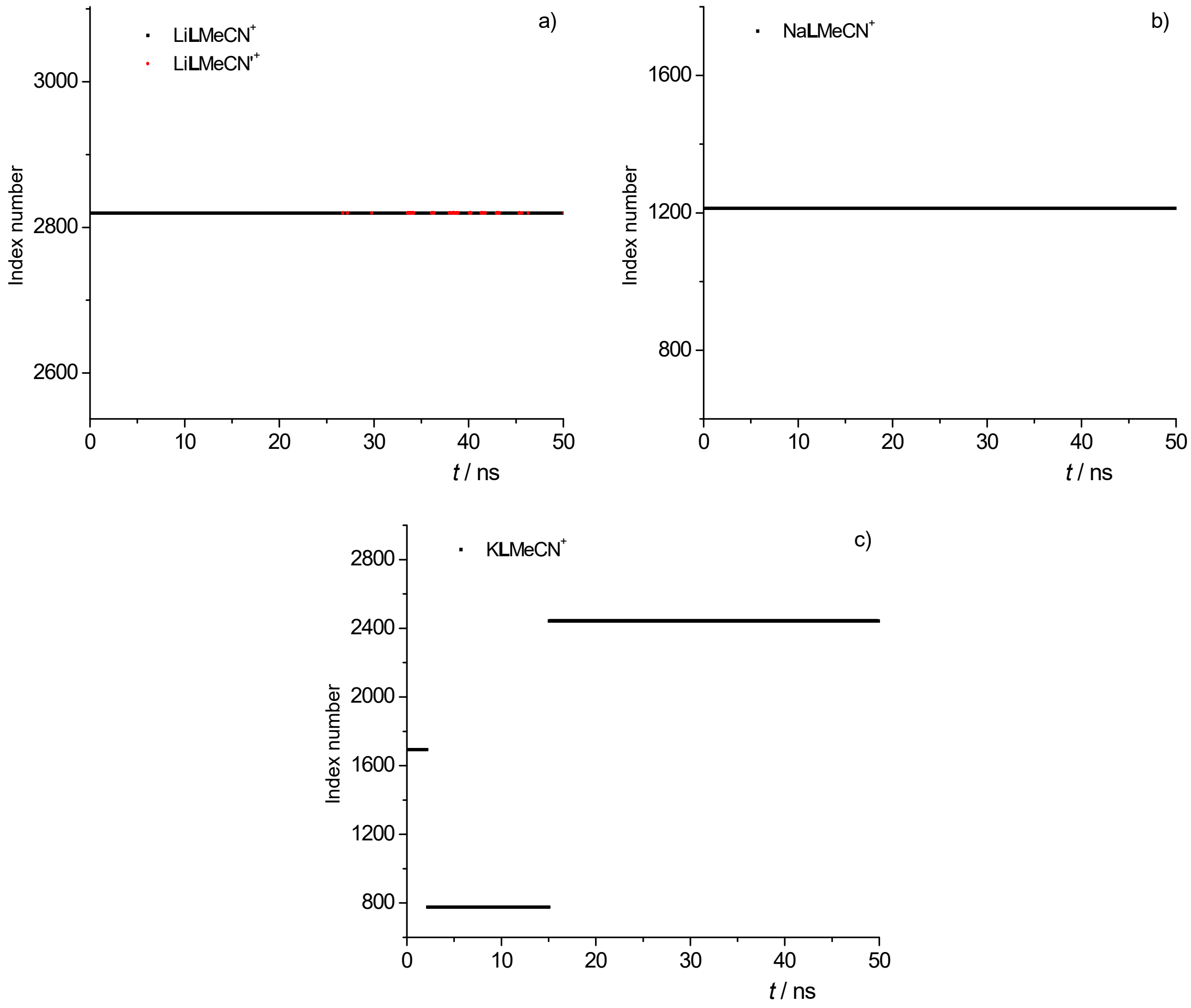

Figure S36. Index number of acetonitrile molecules that occupy hydrophobic cavities of a) lithium, b) sodium, and c) potassium complexes of **L** during MD simulations in acetonitrile at 25 °C.

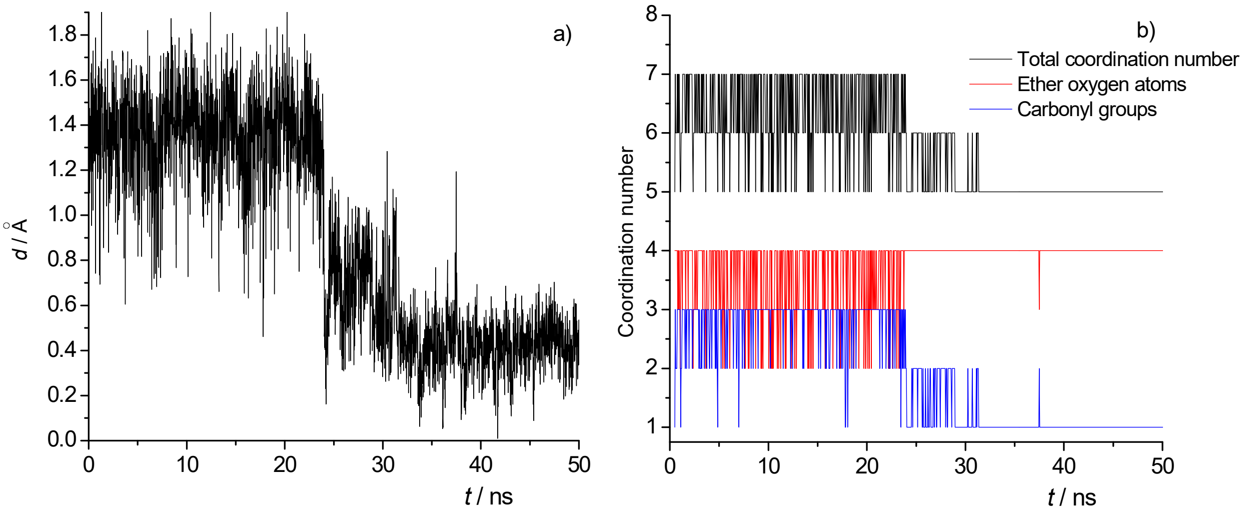

Figure S37. a) Distance between the lithium cation and the geometric center of phenol oxygen atoms and b) coordination number of lithium cation in Li^+–**L** complexes during MD simulations in acetonitrile at 25 °C.

Table S4. Energies of interactions of **L** with lithium, sodium, potassium and rubidium cations and acetonitrile, occurrence time ratio of different chemical species, and the number of carbonyl groups which coordinate metal cations in the complexes obtained by MD simulations in acetonitrile at 25 °C; d_{ref} = 7,85 Å.

	Li^+		Na^+	K^+	Rb^+
	Li**L**MeCN$^+$	Li**L**MeCN$'^+$	Na**L**MeCN$^+$	K**L**MeCN$^+$	Rb**L**MeCN$^+$
$E(M^+–\mathbf{L})$ / kJ mol^{-1}	–570	–518	–514	–412	–342
$E(\mathbf{L}–\mathrm{MeCN})$ / kJ mol^{-1}	–1298	–1349	–1342	–1376	–1254
$E(\mathbf{L}–\mathrm{MeCN_{incl}})$ / kJ mol^{-1}	–53	–11	–53	–52	–51
$E(M^+–\mathrm{MeCN})$ / kJ mol^{-1}	–1	–45	–11	–4	–6
$E(M^+–\mathrm{MeCN_{incl}})$ / kJ mol^{-1}	8	–39	7	5	4
t_{total} / ns	50		50	50	50
t / t_{total}	0.949	0.051	1.00	0.998	1.00
N(coordination, ether-O)	3.76	4.00	4.00	4.00	3.94
N(coordination, C=O)	1.98	1.00	2.89	3.10	2.28
N(coordination, N2)	0	0	0	0	0.24
N(coordination, N3)	0	0	0	0	0
$N(\mathrm{MeCN_{incl}})$	1	1	1	3	1
$\bar{d}$ / Å	7.94	7.89	7.97	7.86	7.85
	8.10	8.37	7.93	7.91	7.85
$\sigma(d)$ / Å	0.29	0.31	0.26	0.25	0.25
	0.35	0.32	0.26	0.25	0.25
$\lvert d - d_{ref} \rvert$ / Å	0.24	0.25	0.23	0.20	0.20
	0.34	0.53	0.21	0.21	0.20

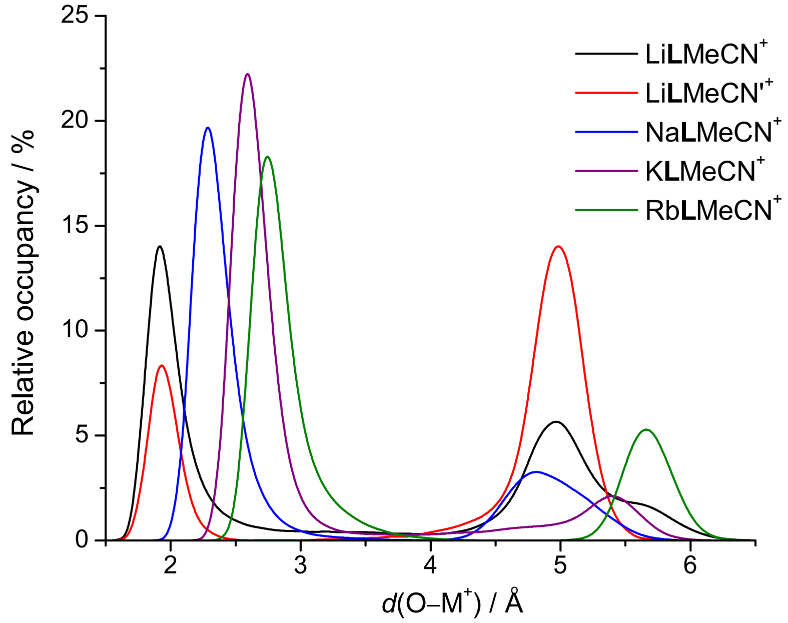

Figure S38. Distribution of metal cation-carbonyl oxygen distances for ML⁺ complexes in acetonitrile obtained by MD simulations. Data was binned at 0.1 Å interval.

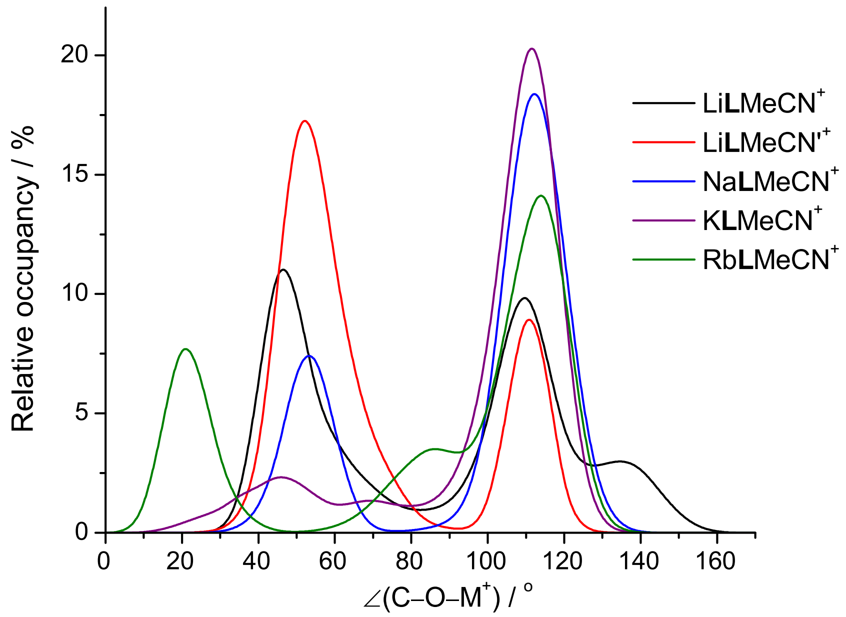

Figure S39. Distribution of metal cation-carbonyl oxygen-carbonyl carbon angle for ML⁺ complexes in acetonitrile obtained by MD simulations. Data was binned at 5° interval.

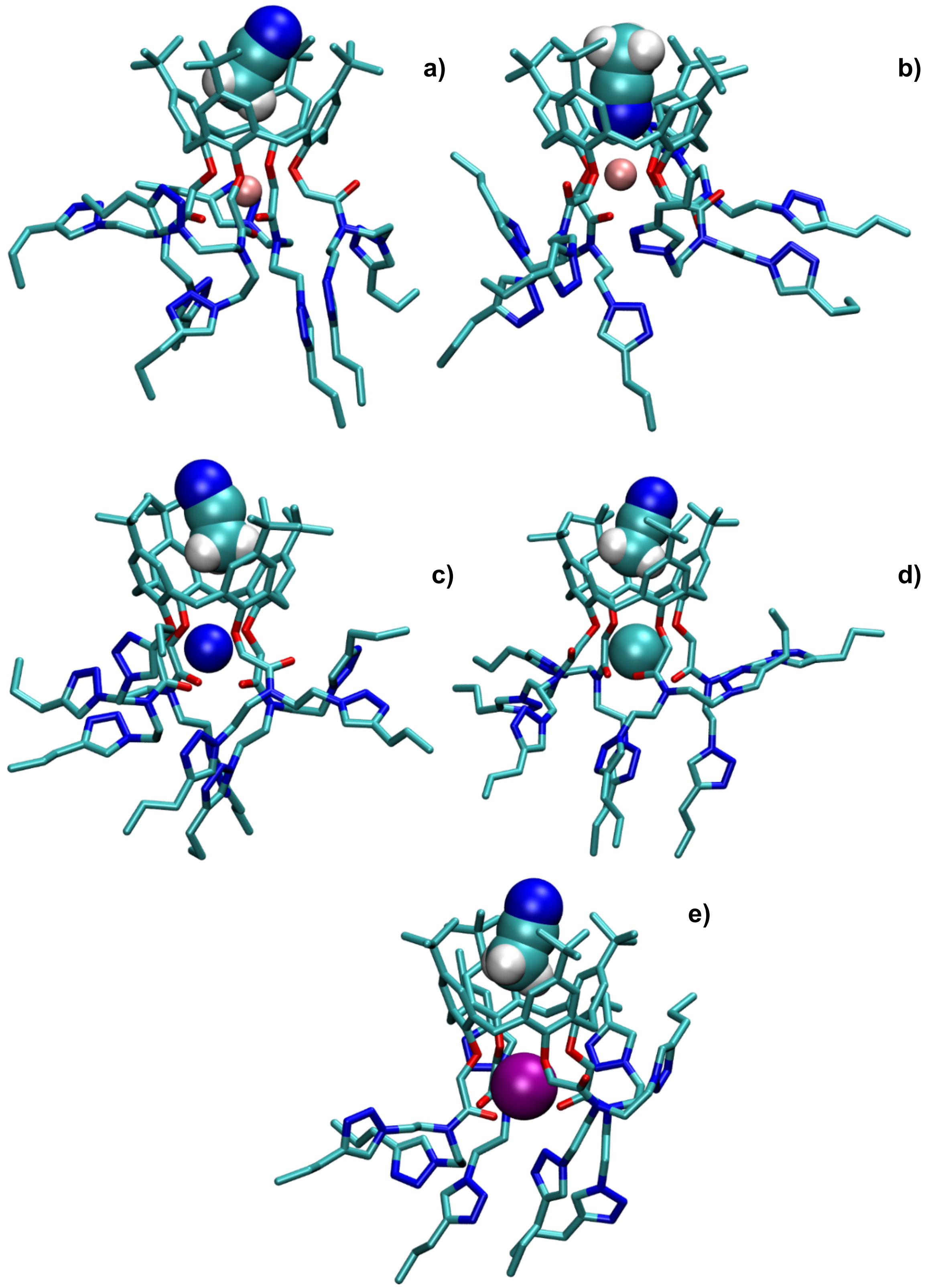

Figure S40. Structures of a) Li**L**MeCN$^+$, b) Li**L**MeCN$'^+$, c) Na**L**MeCN$^+$, d) K**L**MeCN$^+$, and e) Rb**L**MeCN$^+$ adducts obtained by MD simulations at 25 °C. Hydrogen atoms of **L** are omitted for clarity.

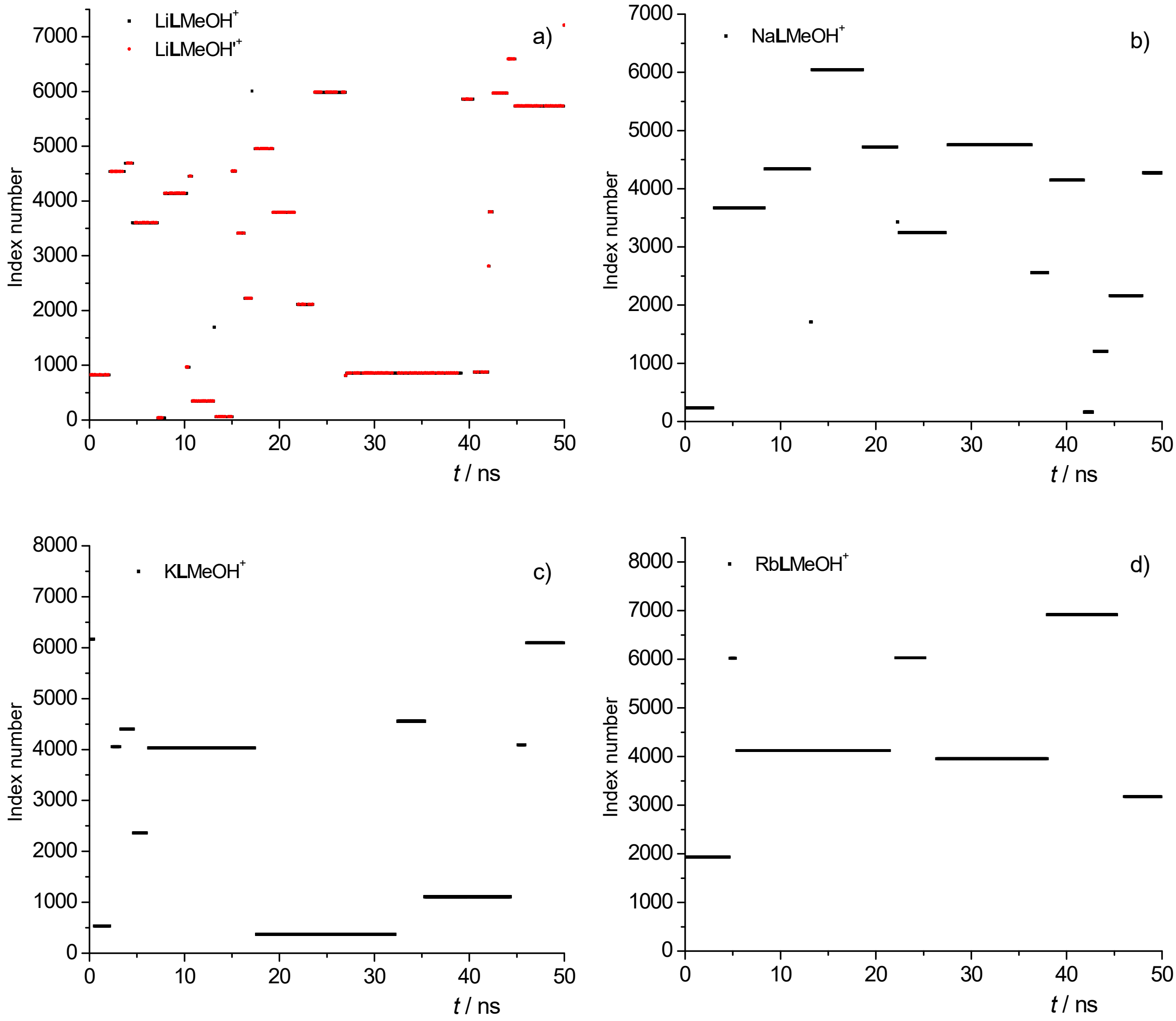

Figure S41. Index number of methanol molecules that occupy hydrophobic cavities of a) lithium, b) sodium, c) potassium, and d) rubidium complexes of **L** during MD simulations in methanol at 25 °C.

Table S5. Energies of interactions of **L** with lithium and sodium cations and methanol, occurrence time ratio of different chemical species, and the number of carbonyl groups which coordinate metal cations in the complexes obtained by MD simulations in methanol at 25 °C; d_{ref} = 7,85 Å.

	Li^+			Na^+	
	Li**L**MeOH$^+$	Li**L**MeOH$'^+$	Li**L**$^+$	Na**L**MeOH$^+$	Na**L**$^+$
E(M^+–**L**) / kJ mol^{-1}	–528	–524	–527	–518	–516
E(**L**–MeOH) / kJ mol^{-1}	–1485	–1458	–1446	–1418	–1389
E(**L**–$MeOH_{incl}$) / kJ mol^{-1}	–50	–29	–	–48	–
E(M^+–MeOH) / kJ mol^{-1}	11	–13	3	3	–2
E(M^+–$MeOH_{incl}$) / kJ mol^{-1}	8	–18	–	7	–
t_{total} / ns		50		50	
t / t_{total}	0.81	0.13	0.06	0.95	0.05
N(coordination, ether-O)	4.00	4.00	4.00	4.00	4.00
N(coordination, C=O)	1.00	1.00	1.00	2.86	2.83
N(coordination, N2)	0	0	0	0	0
N(coordination, N3)	0	0	0	0	0
N($MeOH_{incl}$)	29	27	–	15	–
$\bar{d}$ / Å	7.81	7.78	7.31	7.84	7.48
	8.37	8.38	8.61	8.08	8.22
$\sigma(d)$ / Å	0.28	0.29	0.49	0.27	0.52
	0.30	0.30	0.37	0.28	0.42
$\lvert d - d_{ref} \rvert$ / Å	0.23	0.24	0.59	0.22	0.49
	0.52	0.54	0.77	0.29	0.46

Table S6. Energies of interactions of **L** with potassium and rubidium cations and methanol, occurrence time ratio of different chemical species, and the number of carbonyl groups which coordinate metal cations in the complexes obtained by MD simulations in methanol at 25 °C; d_{ref} = 7,85 Å.

	K^+		Rb^+		Rb^+–MeOH	
	K**L**MeOH$^+$	K**L**$^+$	Rb**L**MeOH$^+$	Rb**L**$^+$	Rb**L**MeOH$^+$	Rb**L**$^+$
E(M$^+$–**L**) / kJ mol^{-1}	–407	–406	–344	–376	–336	–314
E(**L**–MeOH) / kJ mol^{-1}	–1528	–1527	–1488	–520	–1571	–1592
E(**L**–MeOH$_{incl}$) / kJ mol^{-1}	–47	–	–44	–	–44	–
E(M$^+$–MeOH) / kJ mol^{-1}	7	2	5	8	–14	–38
E(M$^+$–MeOH$_{incl}$) / kJ mol^{-1}	6	–	5	–	5	–
t_{total} / ns	50		5.3		44.7	
t / t_{total}	0.96	0.04	0.95	0.05	0.95	0.05
N(coordination, ether-O)	4.00	4.00	4.00	4.00	4.00	4.00
N(coordination, C=O)	2.97	2.94	2.62	2.52	1.00	2.86
N(coordination, N2)	0	0	0.03	0	0	0
N(coordination, N3)	0	0	0.04	0	0	0
N(MeOH$_{incl}$)	11	–	4	–	8	–
$\bar{d}$ / Å	7.87	7.60	7.83	7.66	7.86	7.41
	7.89	7.93	7.87	7.73	7.87	8.02
$\sigma(d)$ / Å	0.27	0.57	0.27	0.69	0.27	0.65
	0.27	0.54	0.27	0.74	0.27	0.60
$\lvert d - d_{ref} \rvert$ / Å	0.21	0.49	0.22	0.58	0.22	0.62
	0.22	0.43	0.21	0.59	0.22	0.51

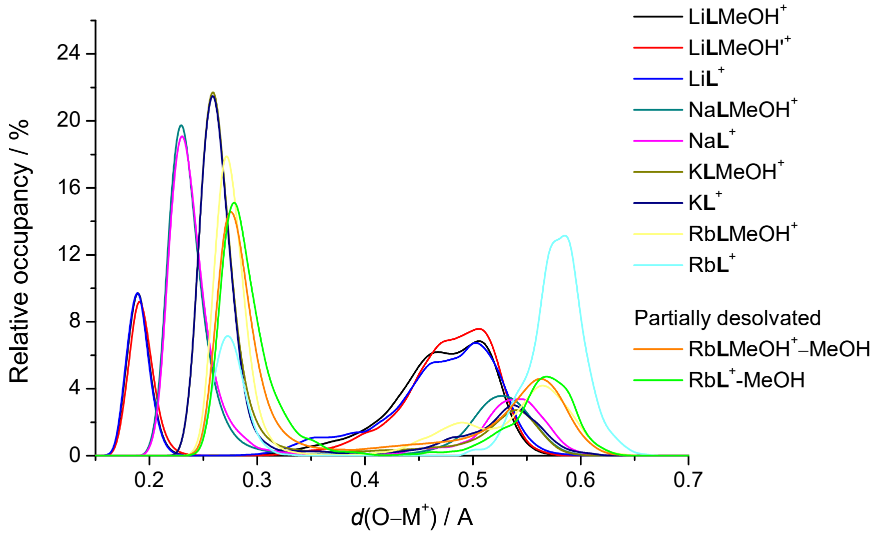

Figure S42. Distribution of metal cation-carbonyl oxygen distances for ML^+ complexes in methanol obtained by MD simulations. Data was binned at 0.1 Å interval.

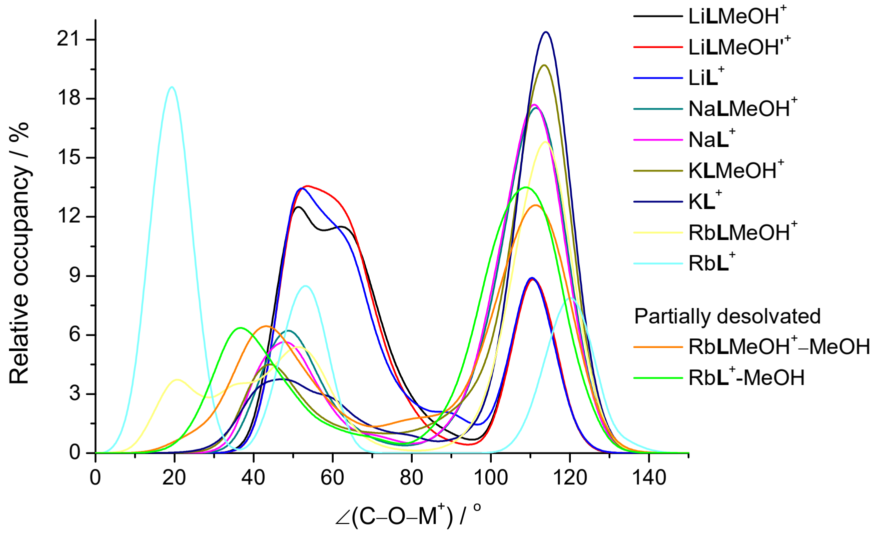

Figure S43. Distribution of metal cation-carbonyl oxygen-carbonyl carbon angle for ML^+ complexes in methanol obtained by MD simulations. Data was binned at 5° interval.

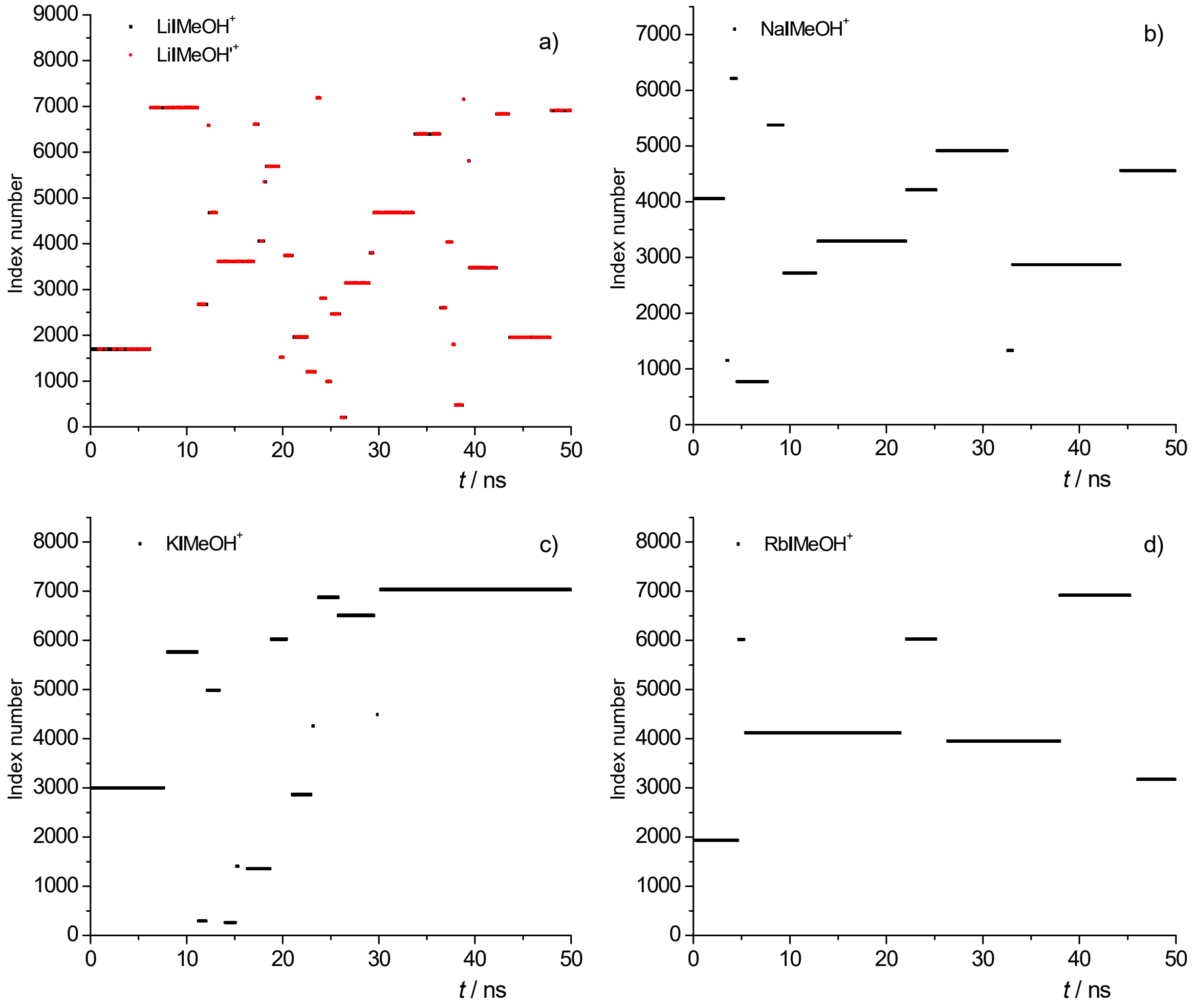

Figure S44. Index number of methanol molecules that occupy hydrophobic cavities of a) lithium, b) sodium, c) potassium, and d) rubidium complexes of **1** during MD simulations in methanol at 25 °C.

Table S7. Energies of interactions of **1** with lithium and sodium cations and methanol, occurrence time ratio of different chemical species, and the number of carbonyl groups which coordinate metal cations in the complexes obtained by MD simulations in methanol at 25 °C; d_{ref} = 7,85 Å.

	Li^+			Na^+	
	Li**1**MeOH$^+$	Li**1**MeOH$'^+$	Li**1**$^+$	Na**1**MeOH$^+$	Na**1**$^+$
E(M$^+$–**1**) / kJ mol^{-1}	–512	–506	–508	–513	–489
E(**1**–MeOH) / kJ mol^{-1}	–2519	–2491	–2472	–2472	–2643
E(**1**–MeOH$_{incl}$) / kJ mol^{-1}	–55	–34	–	–49	–
E(M$^+$–MeOH) / kJ mol^{-1}	2	–23	–5	–5	–4
E(M$^+$–MeOH$_{incl}$) / kJ mol^{-1}	7	–20	–	6	–
t_{total} / ns		50		50	
t / t_{total}	0.73	0.20	0.07	0.96	0.04
N(coordination, ether-O)	4.00	4.00	4.00	4.00	4.00
N(coordination, C=O)	1.05	1.00	1.00	2.79	2.48
N(coordination, N2)	0	0	0	0	0
N(coordination, N3)	0	0	0	0	0
N(MeOH$_{incl}$)	32	32	–	13	–
$\bar{d}$ / Å	7.90	7.90	7.56	7.93	7.60
	8.25	8.26	8.41	7.99	8.14
$\sigma(d)$ / Å	0.30	0.29	0.47	0.27	0.55
	0.30	0.30	0.40	0.28	0.50
$\lvert d - d_{ref}\rvert$ / Å	0.24	0.23	0.43	0.22	0.48
	0.43	0.43	0.59	0.25	0.46

Table S8. Energies of interactions of **1** with potassium and rubidium cations and methanol, occurrence time ratio of different chemical species, and the number of carbonyl groups which coordinate metal cations in the complexes obtained by MD simulations in methanol at 25 °C; d_{ref} = 7,85 Å.

	K^+		Rb^+	
	K**1**MeOH$^+$	K**1**$^+$	Rb**1**MeOH$^+$	Rb**1**$^+$
E(M^+–**1**) / kJ mol^{-1}	–376	–377	–346	–344
E(**1**–MeOH) / kJ mol^{-1}	–2631	–2498	–2631	–2617
E(**1**–$MeOH_{incl}$) / kJ mol^{-1}	–59	–	–46	–
E(M^+–MeOH) / kJ mol^{-1}	–4	–4	3	0
E(M^+–$MeOH_{incl}$) / kJ mol^{-1}	5	–	5	–
t_{total} / ns	50		50	
t / t_{total}	0.90	0.10	0.94	0.06
N(coordination, ether-O)	3.99	3.99	4.00	3.99
N(coordination, C=O)	1.93	1.90	1.93	1.88
N(coordination, N2)	0.20	0.29	0.47	0.36
N(coordination, N3)	0.01	0	0.93	0.95
N($MeOH_{incl}$)	15	–	8	–
$\bar{d}$ / Å	7.86	7.50	7.78	7.31
	7.89	7.96	7.91	8.09
$\sigma(d)$ / Å	0.27	0.61	0.27	0.54
	0.27	0.57	0.26	0.48
$\lvert d - d_{ref} \rvert$ / Å	0.22	0.56	0.22	0.63
	0.22	0.46	0.21	0.44

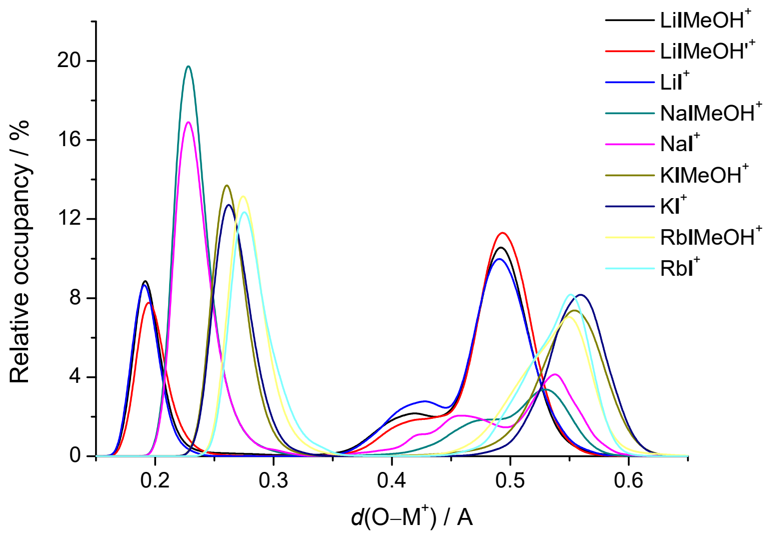

Figure S45. Distribution of metal cation-carbonyl oxygen distances for M^+–**1** complexes in methanol obtained by MD simulations. Data was binned at 0.1 Å interval.

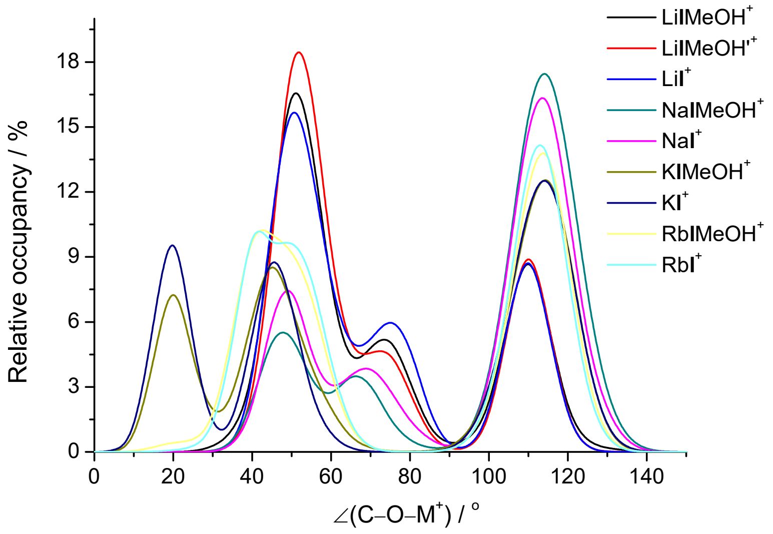

Figure S46. Distribution of metal cation-carbonyl oxygen-carbonyl carbon angle for M^+–**1** complexes in methanol obtained by MD simulations. Data was binned at 5° interval.

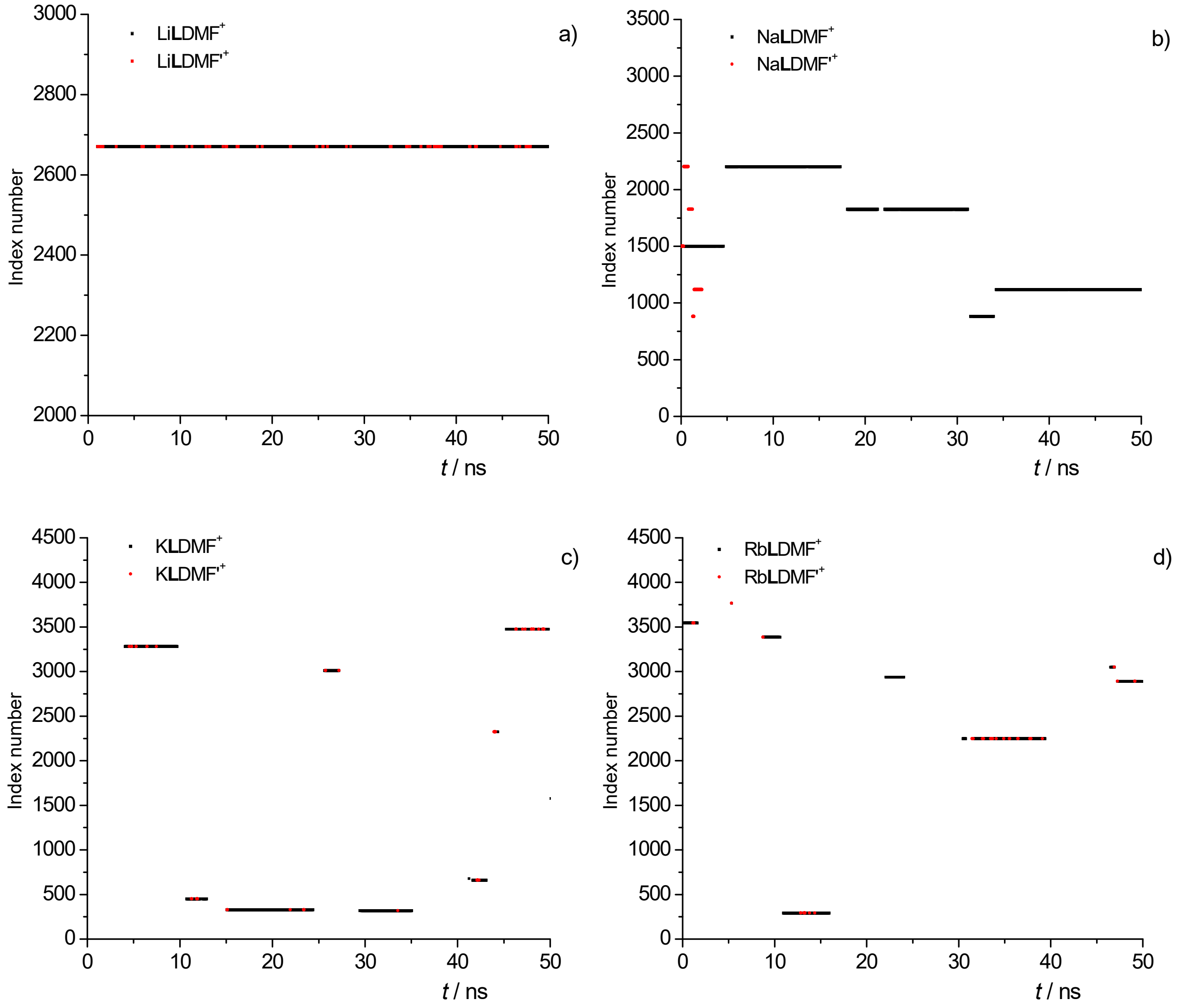

Figure S47. Index number of DMF molecules that occupy hydrophobic cavities of a) lithium, b) sodium, c) potassium, and d) rubidium complexes of **L** during MD simulations in *N,N*-dimethylformamide at 25 °C.

Table S9. Energies of interactions of **L** with lithium and sodium cation and *N,N*-dimethylformamide, occurrence time ratio of different chemical species, and the number of carbonyl groups which coordinate metal cation in the complexes obtained by MD simulations in *N,N*-dimethylformamide at 25 °C; d_{ref} = 7,85 Å.

	Li⁺			Na⁺		
	Li**L**DMF⁺	Li**L**DMF'⁺	Li**L**⁺	Na**L**DMF⁺	Na**L**DMF'⁺	Na**L**⁺
E(M⁺–**L**) / kJ mol⁻¹	–611	–601	–625	–470	–468	–472
E(**L**– DMF) / kJ mol⁻¹	–1468	–1458	–1433	–1576	–1560	–1525
E(**L**– DMF_{incl}) / kJ mol⁻¹	–63	–55	–	–64	–58	–
E(M⁺– DMF) / kJ mol⁻¹	–1	–2	–1	0	–3	–2
E(M⁺– DMF_{incl}) / kJ mol⁻¹	6	4	–	7	5	–
t_{total} / ns		50			50	
t / t_{total}	0.93	0.05	0.02	0.90	0.05	0.05
N(coordination, ether-O)	3.86	3.84	3.72	4.00	4.00	4.00
N(coordination, C=O)	2.62	2.43	2.95	2.02	2.00	2.00
N(coordination, N2)	0	0	0	0	0	0
N(coordination, N3)	0	0	0	0	0	0
N(DMF_{incl})	1	1	–	5	5	–
$\bar{d}$ / Å	7.95	7.79	7.85	8.06	8.11	7.71
	8.33	8.57	7.91	8.24	8.28	8.03
$\sigma(d)$ / Å	0.30	0.28	0.33	0.33	0.46	0.46
	0.31	0.29	0.36	0.33	0.44	0.41
$\lvert d - d_{ref} \rvert$ / Å	0.25	0.23	0.26	0.31	0.40	0.36
	0.51	0.73	0.28	0.43	0.53	0.35

Table S10. Energies of interactions of **L** with potassium and rubidium cations and *N,N*-dimethylformamide, occurrence time ratio of different chemical species, and the number of carbonyl groups which coordinate metal cations in the complexes obtained by MD simulations in *N,N*-dimethylformamide at 25 °C; d_{ref} = 7,85 Å.

	K^+			Rb^+		
	K**L**DMF$^+$	K**L**DMF'$^+$	K**L**$^+$	Rb**L**DMF$^+$	Rb**L**DMF'$^+$	Rb**L**$^+$
E(M$^+$–**L**) / kJ mol^{-1}	–411	–413	–416	–334	–334	–329
E(**L**– DMF) / kJ mol^{-1}	–1520	–1517	–1442	–1376	–1362	–1329
E(**L**– DMF_{incl}) / kJ mol^{-1}	–69	–65	–	–65	–60	–
E(M$^+$– DMF) / kJ mol^{-1}	7	10	5	–1	–3	–4
E(M$^+$– DMF_{incl}) / kJ mol^{-1}	5	3	–	4	3	–
t_{total} / ns		50			50	
t / t_{total}	0.60	0.02	0.39	0.41	0.02	0.57
N(coordination, ether-O)	4.00	4.00	4.00	4.00	4.00	4.00
N(coordination, C=O)	2.95	2.95	2.95	2.22	2.20	2.15
N(coordination, N2)	0	0	0	0.20	0.17	0.14
N(coordination, N3)	0	0	0	0	0	0
N(DMF_{incl})	10	8	–	8	7	–
$\bar{d}$ / Å	8.03	8.09	7.64	8.14	8.24	7.79
	8.07	8.13	7.83	7.96	7.91	7.69
$\sigma(d)$ / Å	0.33	0.48	0.55	0.33	0.45	0.56
	0.33	0.47	0.52	0.33	0.44	0.55
$\lvert d - d_{ref} \rvert$ / Å	0.30	0.45	0.45	0.37	0.52	0.44
	0.32	0.45	0.41	0.27	0.36	0.44

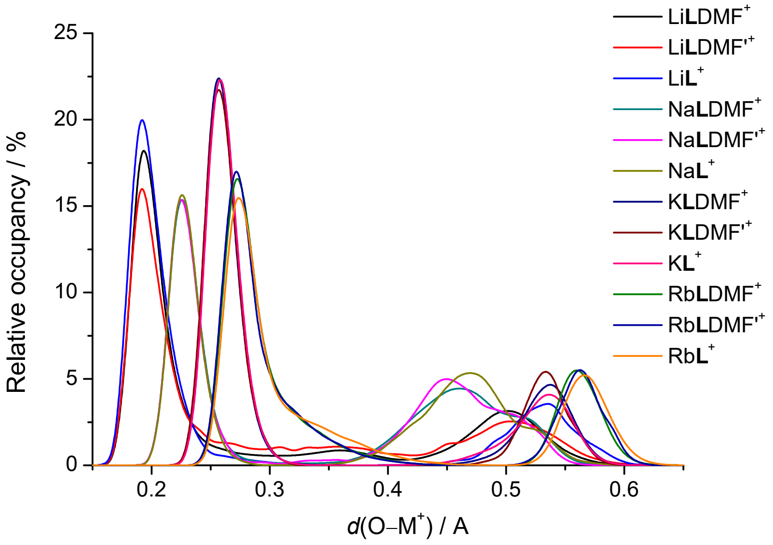

Figure S48. Distribution of metal cation-carbonyl oxygen distances for M^+–**L** complexes in *N,N*-dimethylformamide obtained by MD simulations. Data was binned at 0.1 Å interval.

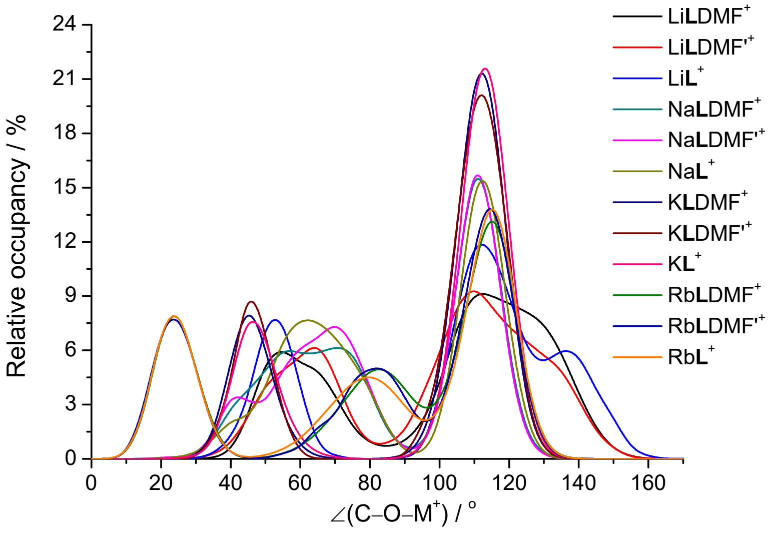

Figure S49. Distribution of metal cation-carbonyl oxygen-carbonyl carbon angle for M^+–**L** complexes in *N,N*-dimethylformamide obtained by MD simulations. Data was binned at 5° interval.

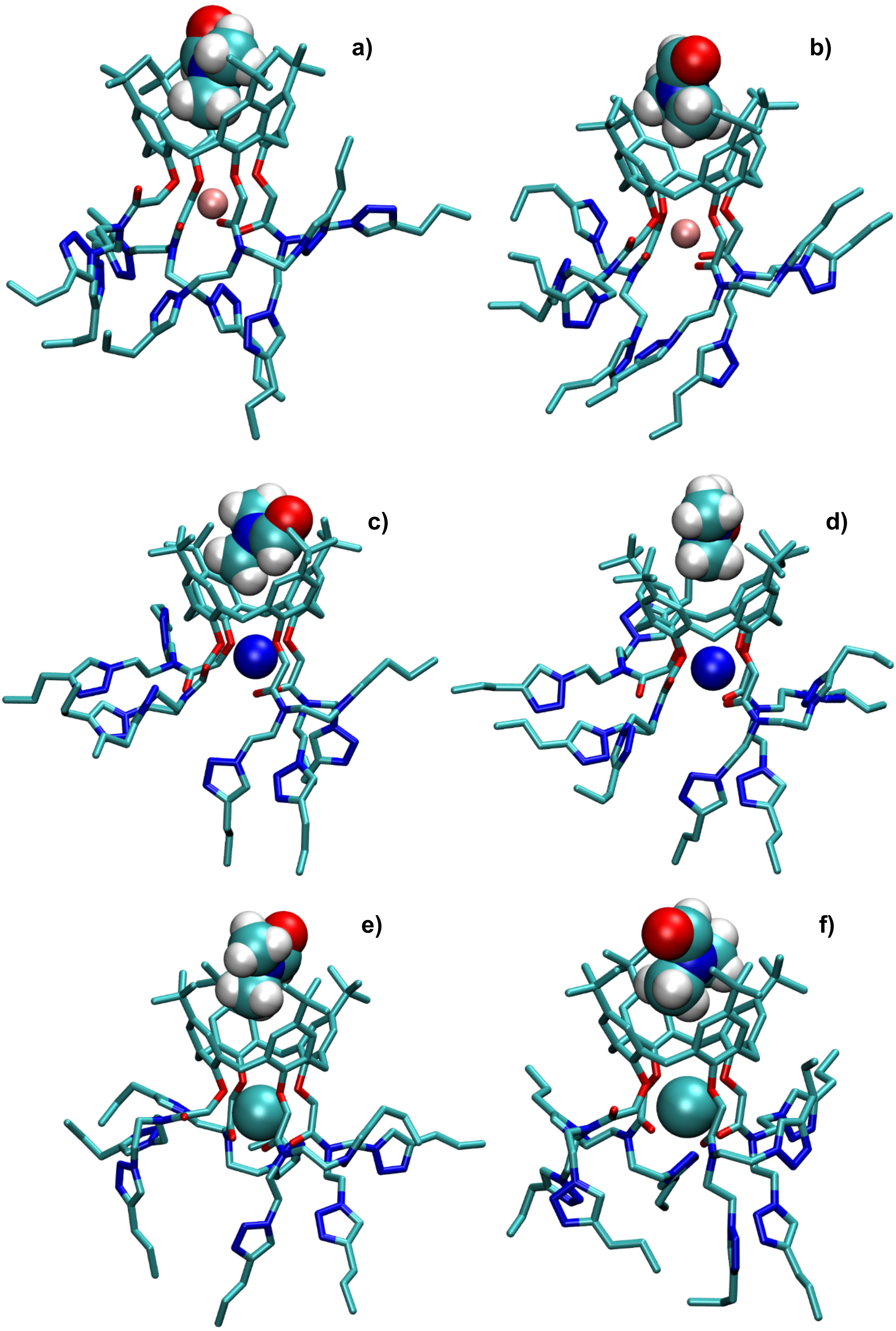
a)
b)
c)
d)
e)
f)

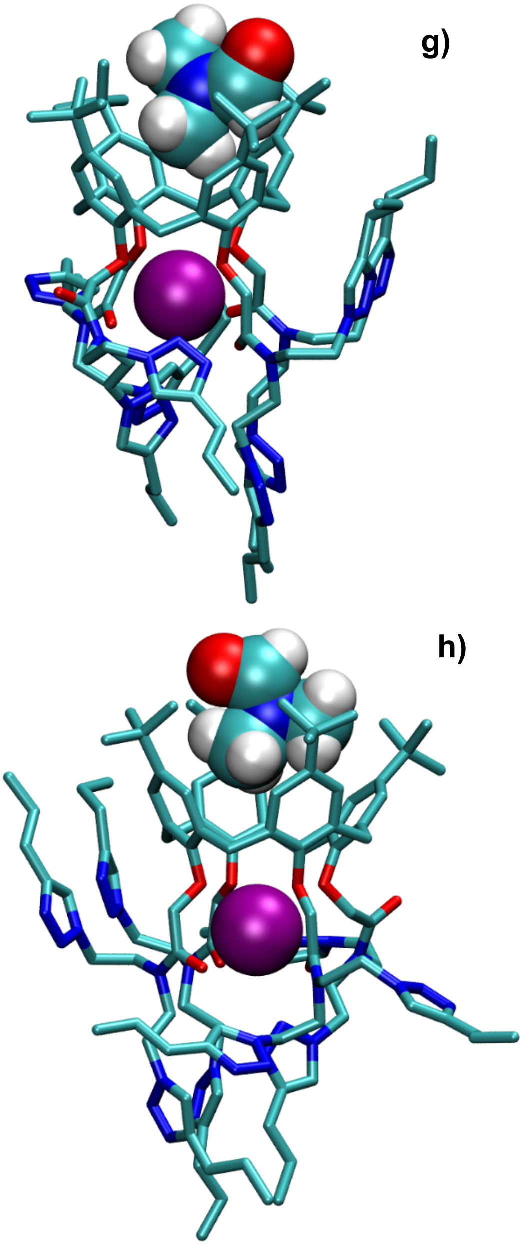

Figure S50. Structures of a) Li**L**DMF^{+}, b) Li**L**DMF$'^{+}$, c) Na**L**DMF^{+}, d) Na**L**DMF$'^{+}$, e) K**L**DMF^{+}, f) K**L**DMF$'^{+}$, g) Rb**L**DMF^{+}, and h) Rb**L**DMF$'^{+}$ adducts obtained by MD simulations at 25 °C. Hydrogen atoms of **L** are omitted for clarity.

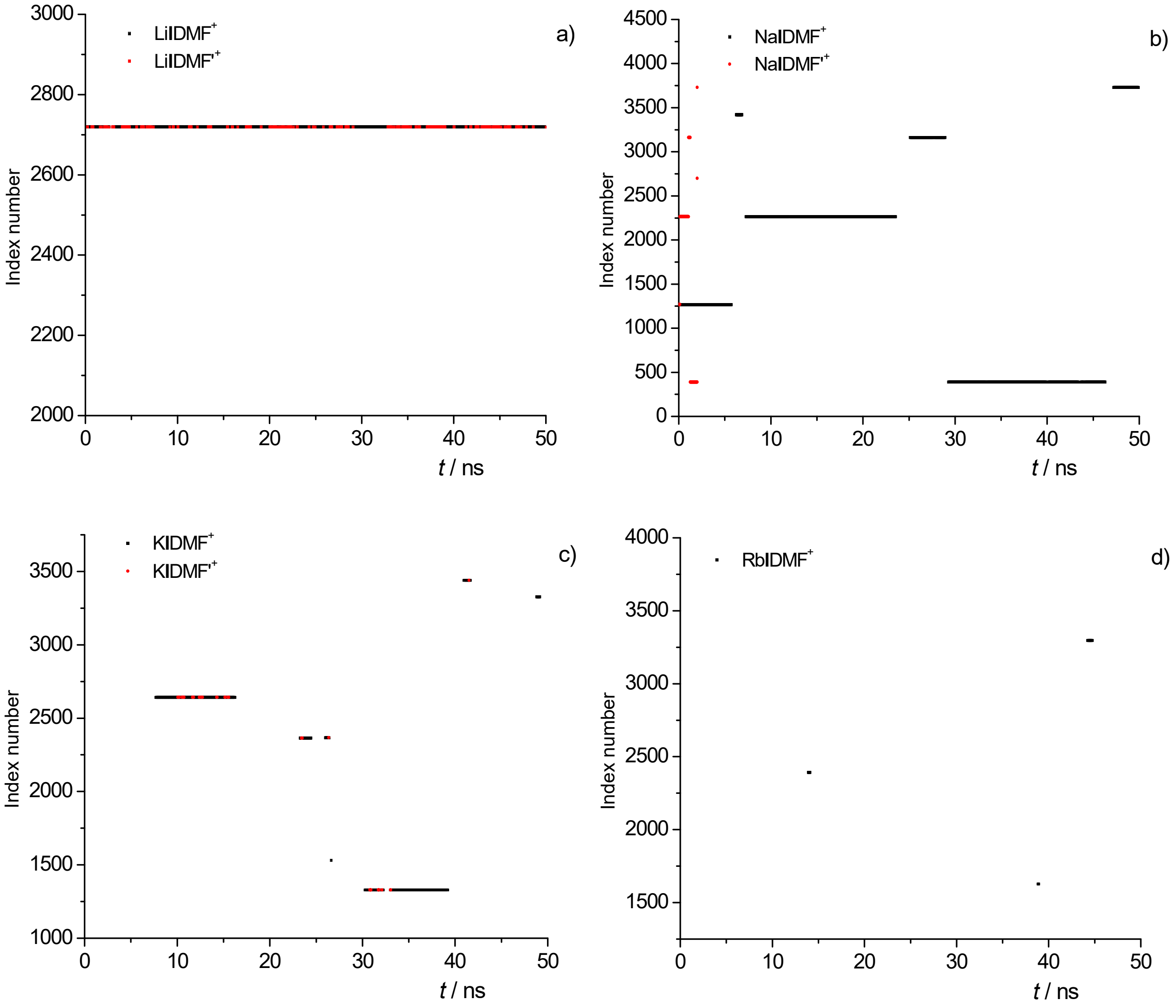

Figure S51. Index number of DMF molecules that occupy hydrophobic cavities of a) lithium, b) sodium, c) potassium, and d) rubidium complexes of **1** during MD simulations in *N*,*N*-dimethylformamide at 25 °C.

Table S11. Energies of interactions of **1** with lithium and sodium cation and *N,N*-dimethylformamide, occurrence time ratio of different chemical species, and the number of carbonyl groups which coordinate metal cation in the complexes obtained by MD simulations in *N,N*-dimethylformamide at 25 °C; d_{ref} = 7,85 Å.

	Li^+			Na^+		
	Li**1**DMF$^+$	Li**1**DMF'$^+$	Li**1**$^+$	Na**1**DMF$^+$	Na**1**DMF'$^+$	Na**1**$^+$
$E(M^+–\mathbf{1})$ / kJ mol^{-1}	–518	–514	–510	–502	–502	–502
$E(\mathbf{1}–$ DMF) / kJ mol^{-1}	–2641	–2682	–2365	–2546	–2522	–2518
$E(\mathbf{1}–$ DMF$_{incl}$) / kJ mol^{-1}	–62	–56	–	–74	–74	–
$E(M^+–$ DMF) / kJ mol^{-1}	–5	–7	6	–9	–9	–13
$E(M^+–$ DMF$_{incl}$) / kJ mol^{-1}	9	6	–	6	3	–
t_{total} / ns		50			50	
t / t_{total}	0.82	0.16	0.02	0.88	0.04	0.08
N(coordination, ether-O)	4.00	4.00	3.72	4.00	4.00	4.00
N(coordination, C=O)	1.33	1.19	2.95	2.80	2.79	2.89
N(coordination, N2)	0	0	0	0	0	0
N(coordination, N3)	0	0	0	0	0	0
N(DMF$_{incl}$)	1	1	–	6	6	–
$\bar{d}$ / Å	8.15	8.11	7.63	8.01	7.90	7.86
	8.23	8.36	8.29	8.25	8.41	7.86
$\sigma(d)$ / Å	0.42	0.54	0.44	0.33	0.39	0.44
	0.41	0.54	0.40	0.34	0.39	0.45
$\lvert d - d_{ref} \rvert$ / Å	0.41	0.47	0.39	0.29	0.31	0.35
	0.47	0.63	0.50	0.44	0.61	0.36

Table S12. Energies of interactions of **1** with potassium and rubidium cations and *N,N*-dimethylformamide, occurrence time ratio of different chemical species, and the number of carbonyl groups which coordinate metal cations in the complexes obtained by MD simulations in *N,N*-dimethylformamide at 25 °C; d_{ref} = 7,85 Å.

	K^+			Rb^+	
	K**1**DMF$^+$	K**1**DMF'$^+$	K**1**$^+$	Rb**1**DMF$^+$	Rb**1**$^+$
$E(M^+–\mathbf{1})$ / kJ mol^{-1}	–374	–374	–374	–349	–330
$E(\mathbf{1}–$ DMF) / kJ mol^{-1}	–2495	–2421	–2503	–2755	–2545
$E(\mathbf{1}–$ DMF$_{incl}$) / kJ mol^{-1}	–61	–64	–	–58	–
$E(M^+–$ DMF) / kJ mol^{-1}	–6	–4	–9	4	–5
$E(M^+–$ DMF$_{incl}$) / kJ mol^{-1}	4	3	–	4	–
t_{total} / ns		50		50	
t / t_{total}	0.37	0.01	0.62	0.015	0.985
N(coordination, ether-O)	3.96	3.98	3.91	4.00	4.00
N(coordination, C=O)	1.89	1.92	1.86	1.96	1.90
N(coordination, N2)	0.15	0.10	0.15	1.00	0.99
N(coordination, N3)	0	0	0	0.63	0.32
N(DMF$_{incl}$)	8	5	–	8	–
$\bar{d}$ / Å	8.02	7.94	7.59	7.65	6.78
	8.07	8.22	7.77	8.29	8.38
$\sigma(d)$ / Å	0.38	0.48	0.81	0.25	0.73
	0.35	0.49	0.79	0.27	0.58
$\|d - d_{ref}\|$ / Å	0.34	0.41	0.73	0.27	1.14
	0.34	0.54	0.67	0.46	0.69

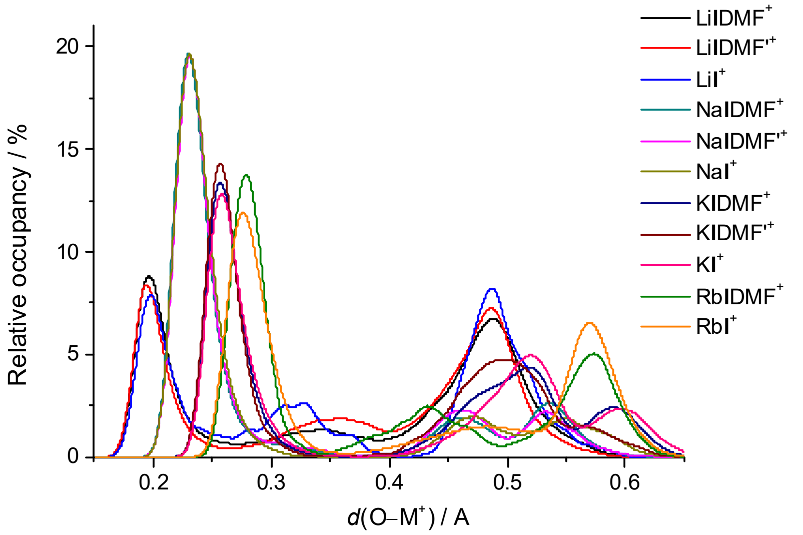

Figure S52. Distribution of metal cation-carbonyl oxygen distances for M^+–**l** complexes in *N*,*N*-dimethylformamide obtained by MD simulations. Data was binned at 0.1 Å interval.

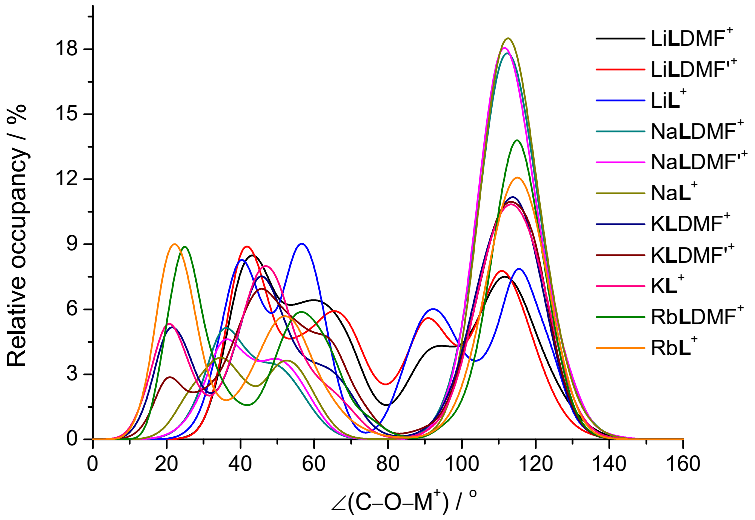

Figure S53. Distribution of metal cation-carbonyl oxygen-carbonyl carbon angle for M^+–**l** complexes in *N*,*N*-dimethylformamide obtained by MD simulations. Data was binned at 5° interval.

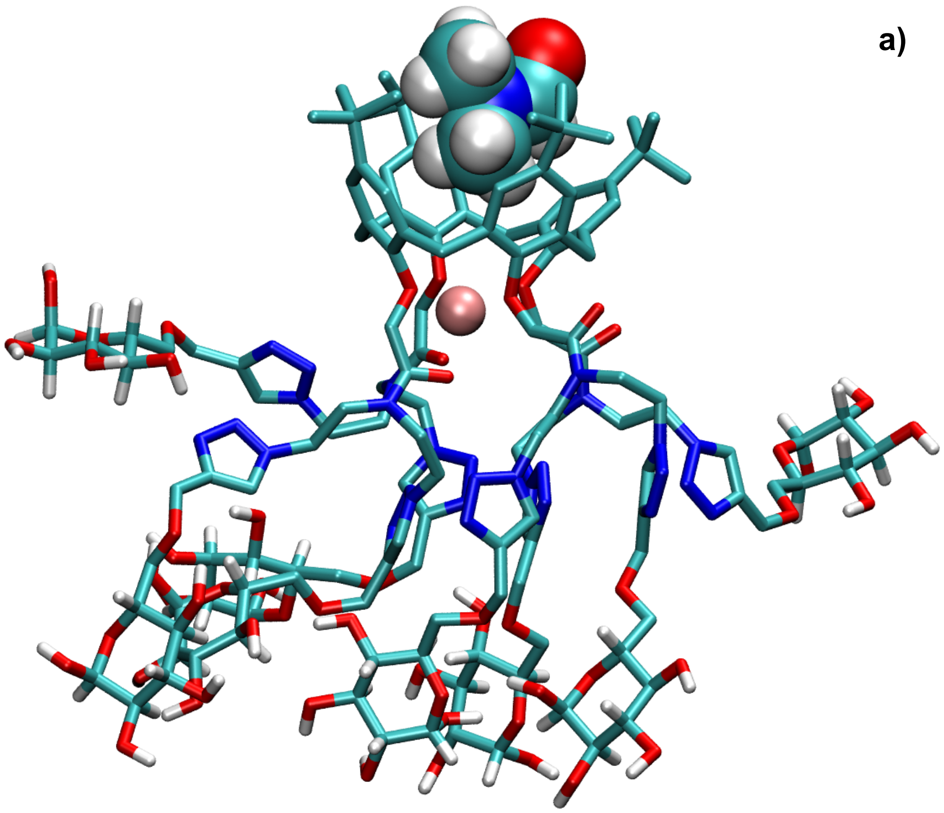
a)

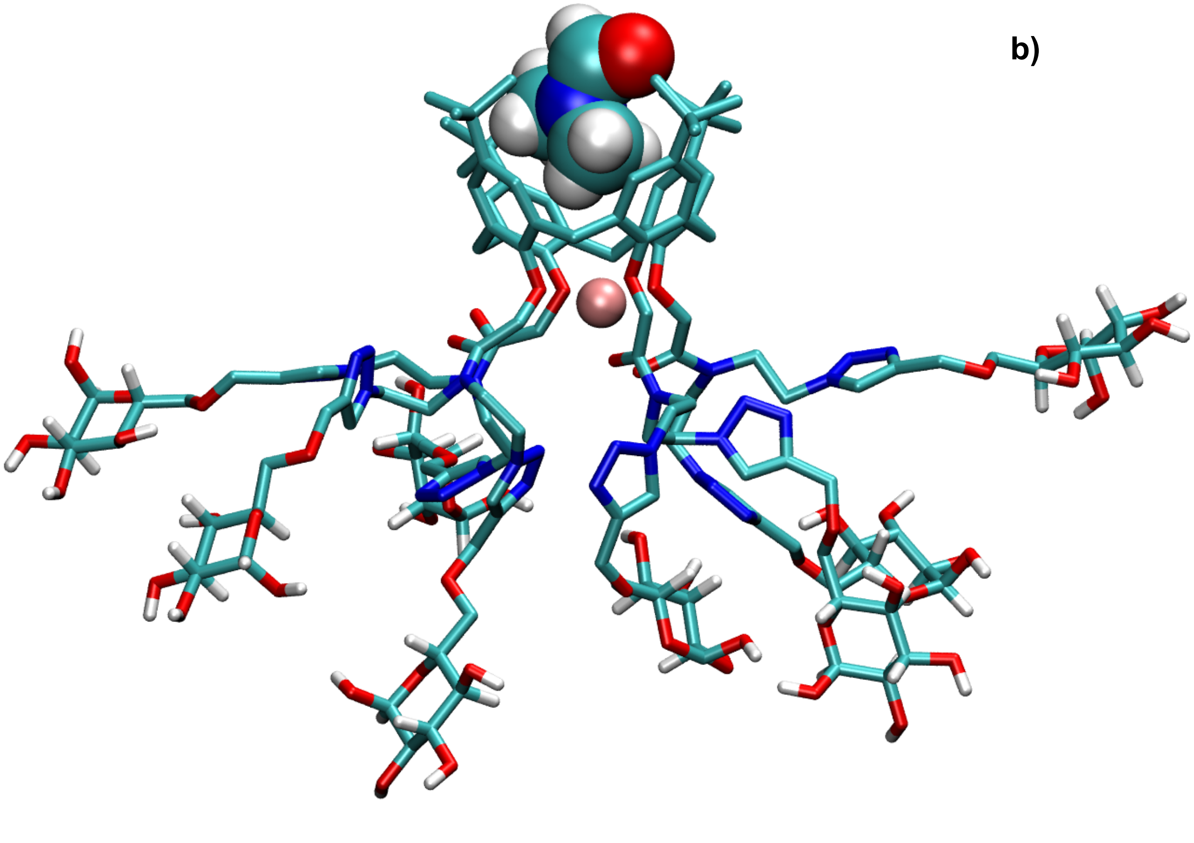
b)

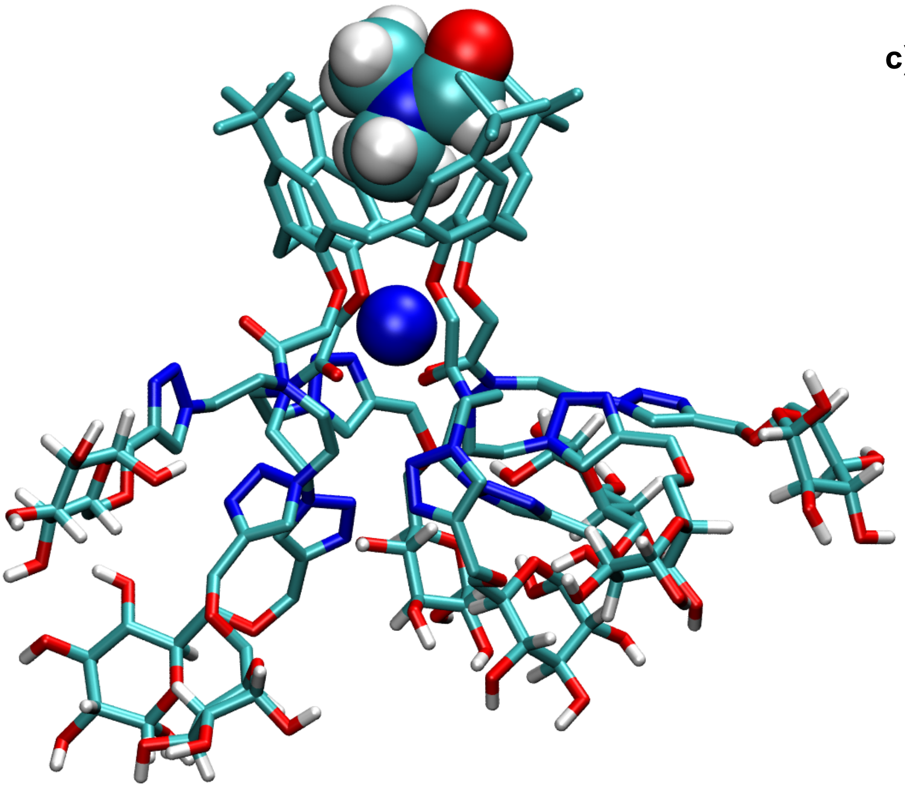

c)

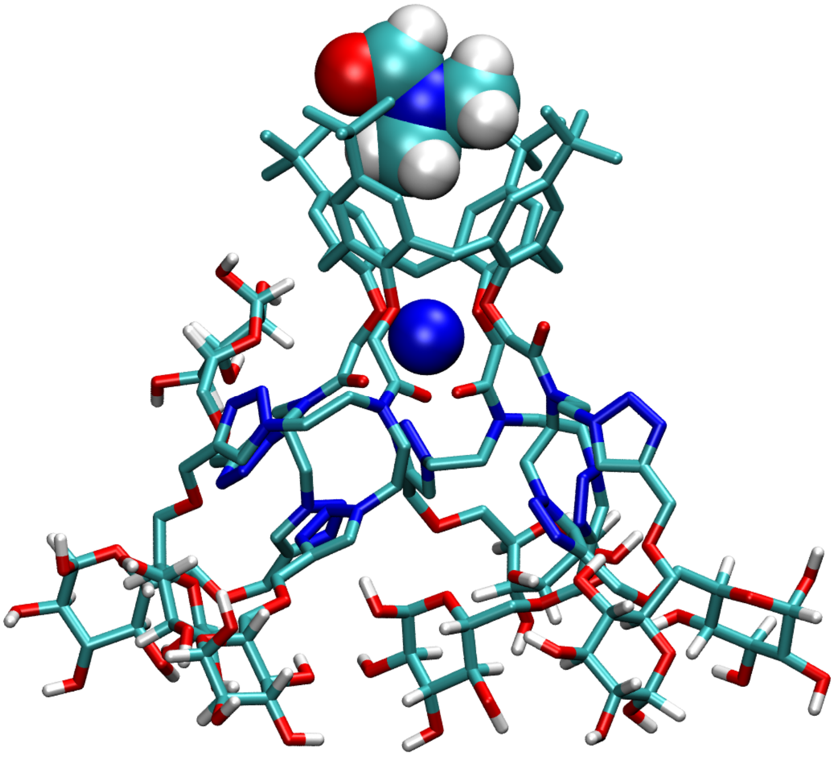

d)

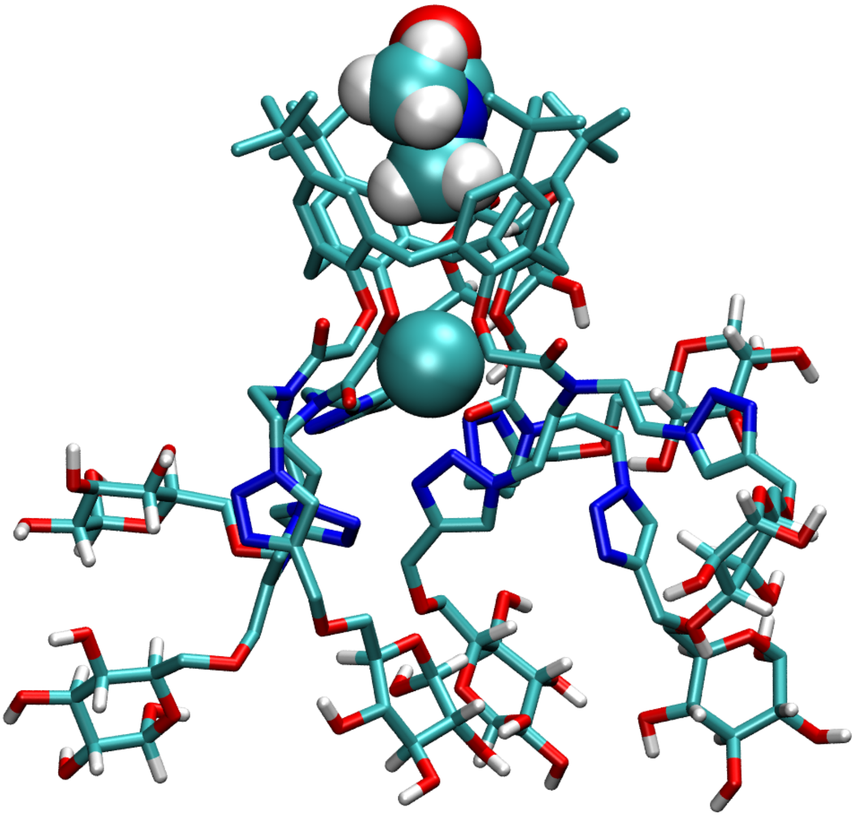

e)

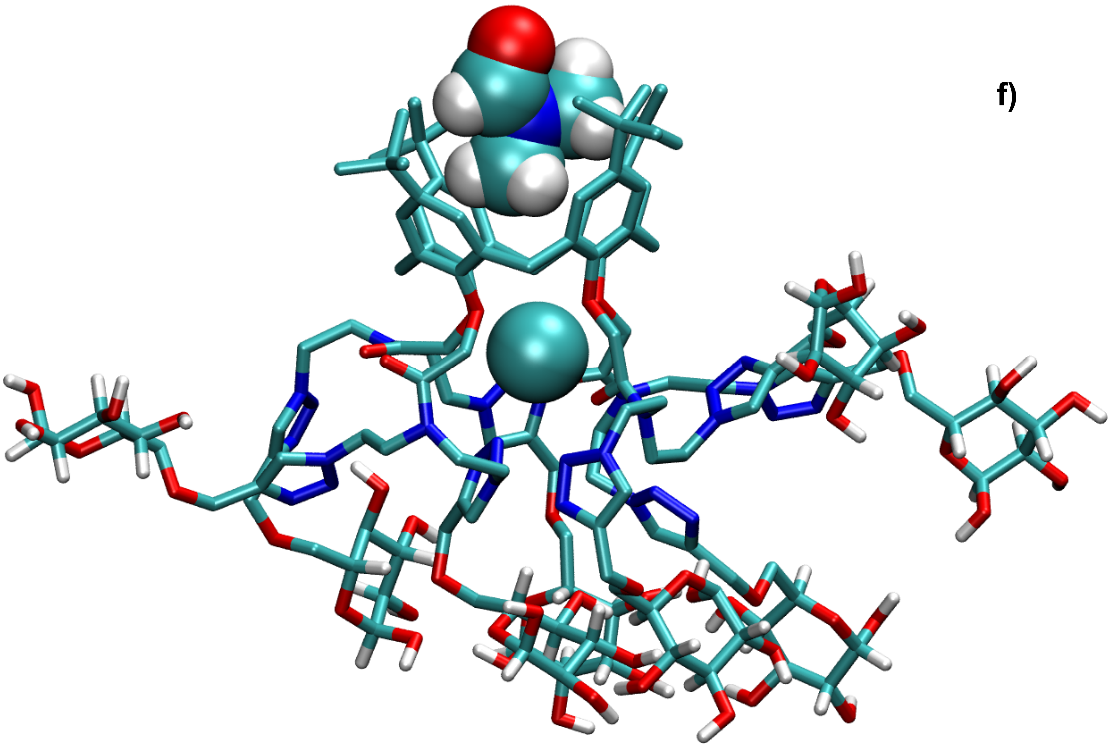

f)

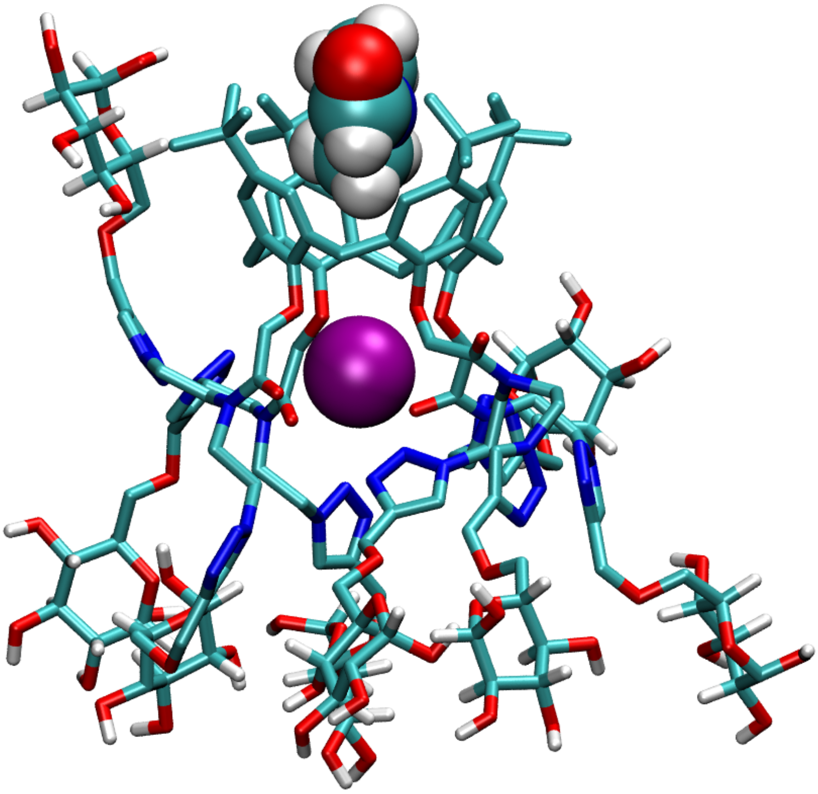

g)

Figure S54. Structures of a) Li**l**DMF$^+$, b) Li**l**DMF$^{\prime+}$, c) Na**l**DMF$^+$, d) Na**l**DMF$^{\prime+}$, e) K**l**DMF$^+$, f) K**l**DMF$^{\prime+}$, and g) Rb**l**DMF$^+$ adducts obtained by MD simulations at 25 °C. Hydrogen atoms of **l** are omitted for clarity.

11. Molecular dynamics simulations: graphical summation of the results obtained for alkali metal complexes with glycocalixarene I

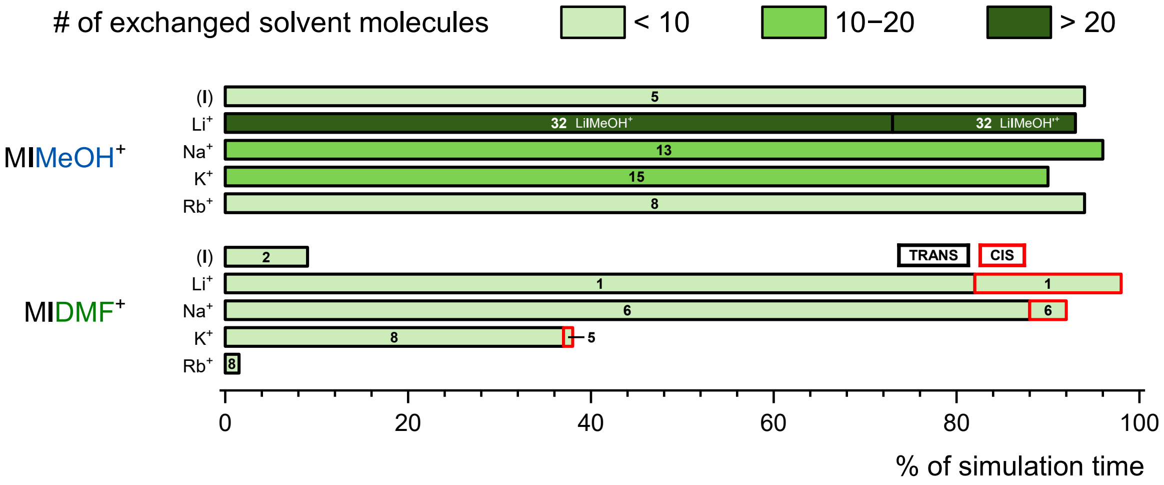

Figure S55. Inclusion of solvent molecules in the hydrophobic cavity of receptor **I** and its complexes observed by MD simulations (t_{tot} = 50 ns). Fractions of the simulation time corresponding to solvent-adduct existance, number of solvent molecules included and their orientation. Species marked by apostrophe denote these in which cations are additionally coordinated by solvent molecules. In the case of DMF, TRANS and CIS marks indicate positions of methyl group relative to oxygen atom.

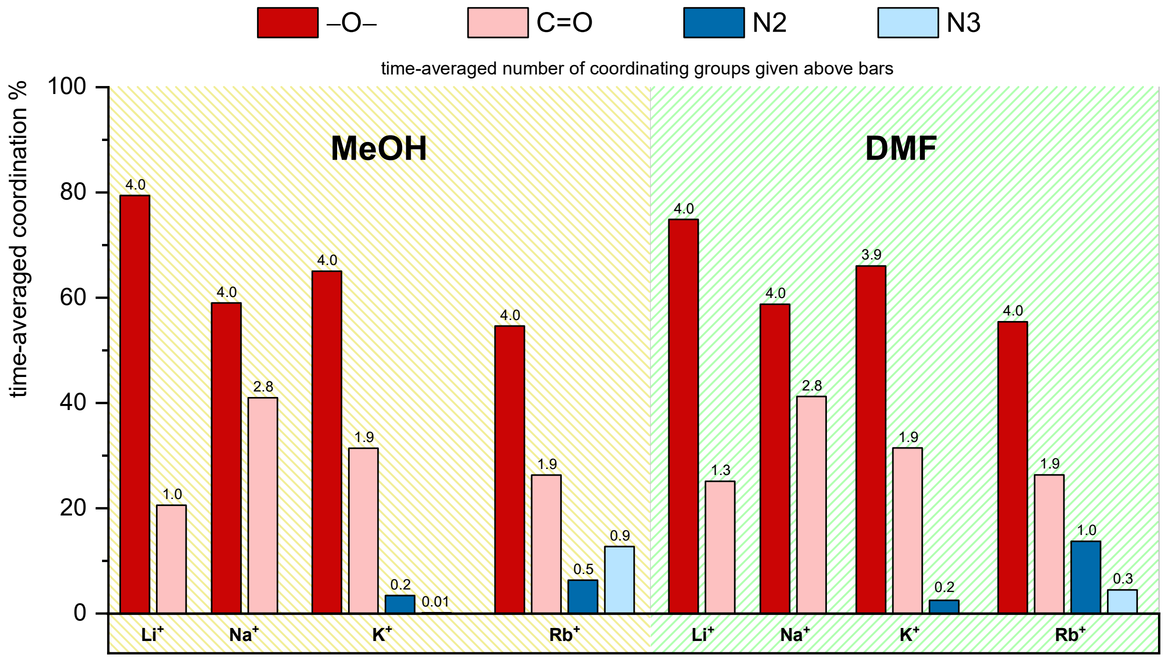

Figure S56. Time-averaged percentage and number of cation-coordinating groups of **1** in MeOH and DMF obtained by MD simulations.
